# Surgery Versus Epilation for the Treatment of Minor Trichiasis in Ethiopia: A Randomised Controlled Noninferiority Trial

**DOI:** 10.1371/journal.pmed.1001136

**Published:** 2011-12-13

**Authors:** Saul N. Rajak, Esmael Habtamu, Helen A. Weiss, Amir Bedri Kello, Teshome Gebre, Asrat Genet, Robin L. Bailey, David C. W. Mabey, Peng T. Khaw, Clare E. Gilbert, Paul M. Emerson, Matthew J. Burton

**Affiliations:** 1 The London School of Hygiene and Tropical Medicine, London, United Kingdom; 2 The Carter Center, Addis Ababa, Ethiopia; 3 Light For The World, Addis Ababa, Ethiopia; 4 The Amhara Regional Health Bureau, Bahir Dar, Ethiopia; 5 NIHR Biomedical Research Centre for Ophthalmology, Moorfields Eye Hospital and UCL Institute of Ophthalmology and UCL Partners AHSC, London, United Kingdom; 6 The Carter Center, Atlanta, United States of America; Kilimanjaro Centre for Community Ophthalmology, Tanzania

## Abstract

**Background:**

Trachomatous trichiasis can cause corneal damage and visual impairment. WHO recommends surgery for all cases. However, in many regions surgical provision is inadequate and patients frequently decline. Self-epilation is common and was associated with comparable outcomes to surgery in nonrandomised studies for minor trichiasis (<six lashes touching eye). This trial investigated whether epilation is noninferior to surgery for managing minor trichiasis.

**Methods and Findings:**

1,300 individuals with minor trichiasis from Amhara Regional State, Ethiopia were recruited and randomly assigned (1∶1) to receive trichiasis surgery or epilation. The epilation group were given new forceps and epilation training. The surgical group received trichiasis surgery. Participants were examined every 6 months for 2 years by clinicians masked to allocation, with 93.5% follow-up at 24 months. The primary outcome measure (“failure”) was ≥five lashes touching the eye or receiving trichiasis surgery during 24 months of follow-up, and was assessed for noninferiority with a 10% prespecified noninferiority margin. Secondary outcomes included number of lashes touching, time to failure, and changes in visual acuity and corneal opacity.

Cumulative risk of failure over 24 months was 13.2% in the epilation group and 2.2% in the surgical group (risk difference = 11%). The 95% confidence interval (8.1%–13.9%) includes the 10% noninferiority margin. Mean number of lashes touching the eye was greater in the epilation group than the surgery group (at 24 months 0.95 versus 0.09, respectively; *p*<0.001); there was no difference in change in visual acuity or corneal opacity between the two groups.

**Conclusions:**

This trial was inconclusive regarding inferiority of epilation to surgery for the treatment of minor trichiasis, relative to the prespecified margin. Epilation had a comparable effect to surgery on visual acuity and corneal outcomes. We suggest that surgery be performed whenever possible but epilation be used for treatment of minor trichiasis patients without access to or declining surgery.

**Trial registration:**

ClinicalTrials.gov NCT00522912

*Please see later in the article for the Editors' Summary*

## Abstract 2

In this randomized, non-inferiority trial, Saul Rajak et al compare epilation and surgery for the management of minor trichiasis in Ethiopia, the country with the most cases of trachomatous trichiasis.

## Editors' Summary

**Background:**

About 40 million people are affected at any one time by active trachoma, an infectious eye disease caused by the bacterium *Chlamydia trachomatis*. Trachoma, which is responsible for more than 3% of the world's blindness, mostly affects people living in rural areas in developing countries where there are water shortages, poor personal hygiene, and crowded living conditions. *C. trachomatis* is spread through contact with infected eye secretions or with contaminated towels or clothes, and by flies. Recurrent infections with *C. trachomatis* during childhood cause inflammation of the lining of the eye lid (chronic conjunctival inflammation), which can lead to conjunctival scarring. If this scarring is severe, the eyelids turn inwards and the eye lashes rub across the eye's surface (the cornea). This condition—trachomatous trichiasis—is extremely painful. Patients describe the pain like having thorns scraping their eyes when they blink. If left untreated, trichiasis can lead to irreversible corneal opacities and visual impairment.

**Why Was This Study Done?:**

The SAFE strategy—surgery for trichiasis, antibiotics for infection, and facial cleanliness and environmental improvements to reduce transmission—aims to control trachoma in countries where it is common. Unfortunately, current surgical activity is only keeping up with new cases of trichiasis; it is not clearing the backlog. The reasons for this treatment gap are complex but in many regions surgical provision is inadequate. Moreover, although the World Health Organization recommends surgery for all cases of trachomatous trichiasis, people with minor trichiasis (only a few eyelashes touching the cornea) often decline surgery, preferring to pull out their eyelashes (epilation), an intervention that has to be repeated when the eyelashes regrow. In this randomized, noninferiority trial, the researchers compare epilation and surgery for the management of minor trichiasis in Ethiopia, the country with the most cases of trachomatous trichiasis. In a randomized trial, randomly chosen groups of patients are given different treatments for a disease and then followed to compare the outcomes of these interventions. A noninferiority trial investigates whether one treatment is not worse than another treatment.

**What Did the Researchers Do and Find?:**

The researchers randomly assigned 1,300 Ethiopians with minor trichiasis to receive surgery or to be given epilation training and good quality epilation forceps. The primary trial outcome was “failure”—five or more lashes touching the eye or receiving trichiasis surgery during the 24-month follow-up period. The researchers decided in advance that epilation would be deemed noninferior to surgery if its failure rate was less than 10% greater than that of surgery (a noninferiority margin of 10%). Secondary outcomes included the number of lashes touching the eye and changes in visual acuity and corneal opacity. The cumulative risk of failure over 24 months was 13.2% in the epilation group and 2.2% in the surgical group, a difference of 11%. The 95% confidence interval for this difference was 8.1%–13.9%. That is, there was a 95% probability that the true failure rate lay within this range. The mean number of lashes touching the eye at 24 months was 0.95 and 0.09 in the epilation and surgery groups, respectively, a significant difference (that is, a difference unlikely to have occurred by chance). Finally, the changes in visual acuity or corneal opacity during the trial were similar in the two groups.

**What Do These Findings Mean?:**

Because the 95% confidence interval for the difference in failure rate of the two interventions included the preset inferiority margin, these findings provide no evidence that epilation is noninferior to surgery for the management of minor trichiasis. That is, statistically speaking, this trial is inconclusive. Thus, if one were to consider only the primary clinical outcome when deciding whether to include epilation in the management of mild trichiasis, one would reject it because this trial indicates that surgery is better than epilation at preventing lashes touching the eye. However, epilation had a comparable effect to surgery on visual acuity and corneal opacity changes and, importantly, in real life, surgical services are likely to remain unacceptable, unavailable, inaccessible, or prohibitively expensive for many people with trachomatous trichiasis in the medium term. The researchers suggest, therefore, that surgery should be performed for minor trachomatous trichiasis whenever possible but that epilation should be considered when surgery is not available or is declined by the patient.

**Additional Information:**

Please access these Web sites via the online version of this summary at http://dx.doi.org/ 10.1371/journal.pmed.1001136.

- An accompanying PLoS Medicine Research Article by Saul Rajak et al. describes another randomized trial undertaken by these researchers that compares the use of absorbable and silk sutures for the surgical treatment of trachomatous trichiasis in Ethiopia
- The World Health Organization has information on trachoma (in several languages), including details of the Alliance for Global Elimination of Trachoma by the year 2020 (GET 2020) and a personal story about blinding trachoma
- The UK National Health Service Choices web site also provides information on trachoma
- Orbis, an international nonprofit organization devoted to blindness prevention and treatment in developing countries, provides information about trachoma
- The International Trachoma Initiative provides detailed personal story about trichiasis surgery in Ethiopia  information about trachoma and a personal story about trichiasis surgery in Ethiopia
- The Global Atlas of Trachoma is an open-access resource on the geographical distribution of trachoma
- Light for the World is a nonprofit organization dedicated to ensuring the rights of persons with disabilities in developing countries, including people in Ethiopia with trachoma

## Introduction

Trachoma is the leading infectious cause of blindness worldwide [1]. Recurrent episodes of ocular *Chlamydia trachomatis* infection in early childhood provoke chronic conjunctival inflammation (active trachoma). This inflammation can lead to conjunctival scarring, which may cause the eyelids to roll in (entropion) and the lashes to touch the surface of the eye (trachomatous trichiasis [TT]). Trichiasis is probably the main risk factor for the development of corneal opacification (CO) and visual impairment in trachoma. However, TT encompasses a very wide spectrum of disease. At one extreme there may be only a few metaplastic eyelashes without entropion. At the other extreme the whole eyelid can be entropic with all eyelashes and even the skin of the upper eyelid in contact with the ocular surface. The risk of developing CO is related to the severity of the trichiasis, and a distinction has been made between minor TT (one to five lashes touching the eye) and major TT (>five lashes touching the eye) [2]–[5].

Trachoma is endemic in around 50 countries, which are striving to control the disease through the implementation of the SAFE strategy: surgery for trichiasis, antibiotics for infection, and facial cleanliness and environmental improvements to reduce transmission [1],[6]. Currently, the World Health Organization (WHO) recommends that all individuals with one or more trichiatic eyelashes should be offered surgery, irrespective of lash location (central or peripheral) or the degree of entropion. The rationale for this recommendation is a practical one: patients with only a minor degree of trichiasis may not be seen again for a long time and there is a risk that the trichiasis will progress and cause visual impairment [6]. There is broad consensus that major TT should be treated surgically. However, for the management of minor TT the reality is often different, with many patients and clinicians preferring to defer surgery until the TT becomes more problematic. In some countries, such as The Gambia, there has been a policy of epilation (repeated removal of lashes with forceps) for minor trichiasis and surgery for major trichiasis, which predated the WHO guidelines [7].

In recent years the number of individuals (mainly children) with active trachoma has declined significantly from 146 million in 1996 to 40 million in 2007, indicating that the A, F, and E components are effective [1],[8]. In contrast, the estimates of the number of people (mainly adults) living with untreated TT have not shown such encouraging declines: 10.6 million in 1996, 7.6 million in 2003, and 8.2 million in 2007 [1],[8]. Available data suggest that current surgical activity is probably keeping up with incident TT but may not be clearing the backlog. For example, in Ethiopia (the country with the most cases of TT) 1.3 million people were estimated to have TT in 2006, but only around 300,000 operations were performed in the last 5 y [1],[9].

The reasons for this treatment gap are complex and multiple. Firstly, there may be insufficient services to address the backlog in many regions. Many of the health workers trained in TT surgery have been lost from programmes, may have conflicting clinical priorities, or do not have the instruments or consumables to perform surgery [10],[11]. Secondly, services may not be accessible to patients. Several studies have shown financial constraints, transport difficulties, and lack of time to be barriers to attending for surgery [7],[12]–[14]. Thirdly, in many regions many patients (often the majority) refuse the offer of surgery, even when major barriers are addressed by the provision of free surgery at community level [7],[13]–[15]. It is likely that in the next 5 y at least, the overwhelming majority of people with TT will not receive surgical treatment, irrespective of whether the TT is minor or major.

In trachoma endemic regions, TT surgery is usually provided by nonophthalmologists who receive about 2-wk training in one of the WHO recommended procedures: bilamellar tarsal rotation (BLTR) or posterior lamellar tarsal rotation (PLTR) [16],[17]. Well-conducted surgery can be a permanent solution. However, trichiasis frequently recurs after surgery, particularly under operational conditions (as compared to clinical trials) where rates as high as 60% recurrence have been reported [3],[18]–[20].

Currently limited longitudinal data are available on which to base recommendations for individuals with minor TT. One cohort study of individuals with TT who refused surgery found that over a 4-y period, 37% progressed from minor to major TT and 5.1% developed new corneal opacity [4]. In a parallel cohort of patients from the same area who had accepted TT surgery, the comparable risks were 41% and 5.2%, respectively [18]. A second longitudinal study also found no difference in risk of developing new corneal opacity between people receiving TT surgery and those declining it, over a 1-y period [21]. Studies from several trachoma endemic regions suggest that the majority of people with TT epilate [7],[22]. Two cross-sectional studies have found that epilation is associated with a reduced risk of corneal opacity [2],[23]. In high-income settings patients with a minor degree of trichiasis, such as metaplastic or misdirected lashes caused by lid margin disease, are often managed by repeated epilation. Epilation is simple for a trained helper to perform. However, it is an ongoing treatment that needs to be repeated when eyelashes regrow and should be conducted using good quality forceps.

In view of the prevalence of unoperated TT, the limited capacity of current surgical services, concerns over programmatic surgical outcomes, and the high rates of refusal, epilation might be an acceptable alternative to surgery for the management of minor TT. In this randomised trial we tested the hypothesis that epilation is noninferior to surgery for the treatment of minor TT.

## Methods

### Ethics Statement

The National Health Research Ethics Review Committee of the Ethiopian Ministry of Science and Technology, the London School of Hygiene and Tropical Medicine Ethics Committee, and Emory University Institutional Review Board approved the trial. Potential participants were provided with both written and oral information in Amharic about the trial. For those agreeing to participate, written informed consent in Amharic was required prior to enrolment. If the participant was unable to read and write, the information sheet and consent form were read to them and their consent recoded by witnessed thumbprint. An independent Data Safety Monitoring Committee reviewed the trial for patient safety and there were no deviations from the original protocol. No interim analyses for efficacy or futility were planned or conducted. The trial protocol is described in Text S1 and the CONSORT statement in Text S3

### Participants

Eligible participants were individuals aged 18 y or over with previously unoperated minor trichiasis (<six trichiatic eyelashes, some of which may have been epilated and were regrowing) who presented during a TT treatment campaign in rural villages in West Gojjam, Amhara Region, Ethiopia from March to June 2008. Exclusion criteria were previous eyelid surgery, medically unfit, or pregnancy (self-reported or clearly evident). The campaigns were advertised in local markets, churches, and schools. Additionally, health extension workers from the subdistricts (*kebele*) in the study area were trained to recognize trichiasis and visited each village in their *kebele* to identify patients. In individuals with bilateral TT, only one eye was randomly designated as the study eye and included in the analysis, although both eyes were treated.

### Baseline Clinical Assessment

The clinical assessments and surgery were performed in government health centres. A field worker administered a questionnaire in Amharic. Height and weight were measured. Unaided and pinhole LogMAR visual acuities were measured at 4 m, using an ETDRS equivalent Tumbling-E LogMAR chart (Hong Kong Low Vision Centre). The testing distance was reduced to 2 or 1 m if necessary. For those unable to read the LogMAR chart at 1 m their visual acuity was tested by counting fingers at 1 m or hand movements at 0.5 m. For visual acuities of counting fingers or less, LogMAR values were attributed: counting fingers, 2.0; hand movements, 2.5; perception of light, 3.0; no perception of light, 3.5. No patients had spectacles for distance correction. In this report we present only unaided visual acuities. Ophthalmic examinations were conducted in a darkened room using 2.5× magnification loupes and a bright torch. A single ophthalmologist (SNR) performed all baseline examinations. The number of lashes touching the eye was counted (“lash burden”) and also subdivided by the part of the eye contacted when looking straight ahead: cornea, lateral conjunctiva, or medial conjunctiva. Clinical evidence of epilation was identified by the presence of broken or newly growing lashes, or areas of absent lashes. Upper lid entropion was graded by assessing the degree of inward rotation of the eyelid margin (Table S1). The degree of conjunctivilisation of the lid margin (anteroplacement of the muco-cutaneous junction) was assessed (Table S1). The degree of corneal scarring was classified using a modified WHO detailed trachoma grading system (FPC) in which the degree of central corneal scarring (CC2) was subdivided into four, to provide more definition (Table S1) [24]. The Simplified WHO Trachoma Grading System corneal opacity measure (CO) is equivalent to CC2 and CC3 [25]. Tarsal conjunctival papillary inflammation, follicles, and scarring were classified using the WHO FPC grading system [24]. Standardised high-resolution digital photographs were taken of each of these features.

### Interventions

Following the baseline clinical assessment, participants were randomised to one of two intervention groups: surgery or epilation. The posterior lamella tarsal rotation procedure was used in all surgery cases [17]. Surgery was performed under local anaesthesia administered by subcutaneous infiltration of the upper eyelid: 2–3 ml of lidocaine 1%, with adrenaline. The lid was then everted and the posterior lamella (tarsal conjunctiva and tarsal plate) incised parallel to and 3 to 4 mm above the lid margin. The posterior and anterior (obicularis oculi and skin) lamellae were separated by blunt dissection. Three sets of 4/0 silk everting sutures (three-eighth circle, 16-mm cutting needles, Mersilk, Ethicon) were placed to externally rotate the lower border of the eyelid. Postoperatively, the operated eye was padded until the next morning and tetracycline eye ointment was self-administered twice a day for 2 wk. Five nurses, who had previously been trained in and were regularly performing TT surgery, performed the surgery in this trial. They were identified as the best surgeons from a larger group of nine during a 2-d standardisation workshop. This training was conducted by an experienced Ethiopian ophthalmologist (ABK), who had contributed to the development of the WHO TT surgeon certification manual [17]. The posterior lamellar tarsal rotation techniques of the five nurses were carefully observed and standardised to ensure that all performed the operation in the same way. The intraoperative quality of surgery was periodically reviewed during the course of the trial.

Individuals randomised to the epilation group were each given two pairs of high quality, machine-manufactured epilation forceps with round edged tips (for corneal safety) and flat, opposing plates to improve grip and reduce the likelihood of breaking lashes (Tweezerman). The patient and an accompanying adult (“epilator”) with good near vision were trained to perform epilation. They observed the trainer epilate one or two lashes, with particular attention paid to removing the lash at its base to minimise broken stubs. The epilator was then observed epilating lashes and given advice on technique.

### Follow-up Clinical Assessments

All participants in the surgery arm were seen 7–10 d postoperatively, at which point the sutures were removed and any complications noted and treated as needed. Several different ophthalmic nurses who did not take part in subsequent follow-ups conducted the 7–10-d suture removal follow-up. Follow-up assessments were conducted for both groups at 6, 12, 18, and 24 mo. On each occasion an ophthalmic examination was performed and digital photographs taken. Visual acuity was measured at 12 and 24 mo. The preoperative, 12-mo, and 24-mo examinations were conducted by a single ophthalmologist (SNR) and the 6- and 18-mo were conducted by a single ophthalmic nurse (EH). Neither of these examiners was involved in performing treatments in this trial. The examiners were standardised to each other and showed strong agreement between these two observers for the presence of trichiasis in a preliminary assessment of 200 eyes (kappa = 0.86). The presence/absence of notching (overcorrection of the central portion of the lid) and conjunctival granuloma were noted.

Any individual who showed evidence of disease progression, defined as five or more trichiatic lashes or corneal changes related to observed lashes at any follow-up examination, was immediately offered surgery (epilation arm) or repeat surgery (surgery arm) to be performed by a senior surgeon. These individuals continued to be followed up according to the trial protocol. Individuals in whom other ophthalmic pathology (e.g., cataract) was detected were referred to the regional ophthalmic services. New epilating forceps were provided as required. At the final follow-up (24 mo), all participants in the epilation arm were offered TT surgery.

### Outcome Measures

The primary outcome measure was the proportion of individuals at any follow-up who had “failed,” defined as either (1) five or more eyelashes touching the globe or (2) a history of surgery performed in the trial eye at any point during the follow-up period (in the case of the surgical arm this would be repeat surgery). The five or more lash threshold for failure was chosen to make it slightly more stringent than the inclusion criteria. A priori defined secondary outcome measures at 12- and 24-mo were: CO, visual acuity, entropion, conjunctival inflammation and symptoms of pain, subjective visual acuity, epiphora, and dryness.

Change in CO was assessed both by direct comparison of the 1- and 2-y photographs with the baseline photograph, and by comparison of the field grading scores. Photographs for each time point were viewed on a computer screen at about 10× magnification by a single masked observer (MJB). They were first graded using the trial grading system (Table S1). Then the 1- and 2-y images were compared side by side with the baseline image, allowing the direct comparison of opacities to assess whether they had changed; these were graded as improved, no change, or worse. The baseline photographic and field CO scores showed good correlation (linear weighted kappa score 0.74; quadratic weighted kappa score 0.87).

### Randomisation, Allocation Concealment, and Masking

Participants were randomly allocated to the epilation or surgery groups using a 1∶1 allocation ratio for each surgeon, using a computer-generated randomisation sequence with random block sizes. Randomisation was stratified by surgeon because of possible intersurgeon variability (each surgeon had a separate sequence). The London-based statistician held the master randomisation lists. The random allocation sequences for each surgeon were concealed in sequentially numbered, sealed, opaque envelopes, which were colour coded for surgeon and placed in separate containers for each surgeon. The person who prepared these envelopes was independent of all other aspects of the trial. Following the baseline examination, participants were allocated to the next available surgeon. A field worker was responsible for implementing the intervention assignment in a dedicated area, separated from those performing the preoperative examinations. The two individuals (SNR, EH) responsible for all the clinical outcome measurements were masked to the allocation. At follow-up, the trichiasis and corneal examination was performed and recorded before the eyelid was everted, so that the examiner was masked to whether surgery had been performed on the tarsal conjunctiva. The posterior lamella tarsal rotation technique used in this study does not involve a skin incision, so there were no external marks of previous surgery that could unmark the observer.

### Statistical Methods

A noninferiority trial design was chosen to investigate epilation in the management of minor trichiasis because of the potential secondary benefits of this intervention (greater acceptability and availability). Epilation is an ongoing treatment, which is performed when a trichiatic lash regrows. As such it is intrinsically less likely to be effective than surgery at always preventing lashes from touching the eye. In a noninferiority trial a slightly reduced clinical efficacy might be accepted if this is balanced by other secondary benefits; in the case of epilation these include greater acceptability and availability in an endemic population compared to surgery. We chose a noninferiority margin (Δ) of 10% at the outset of this trial as we considered this level to balance the considerations of clinical efficacy and secondary benefits.

The sample of 1,300 trial participants provided 90% power to detect noninferiority based on a two-sided 95% CI approach, assuming a 10% loss to follow-up over 2 y, a 5% failure rate in the surgery group, and a Δ of 10% between the epilation and surgery groups respectively [3],[26].

Data were double-entered into an Access (Microsoft) database and transferred to Stata 11 (StataCorp). Data were analysed by intention to treat. For participants who had bilateral treatment only the randomly designated “study eye” was included in the analysis. Patients were excluded from a specific analysis if they had missing data relevant to that analysis, as indicated in the results. Missing data were not imputed.

The 95% CI for the difference in the proportion of “failures” (proportion having ≥five lashes touching the eye or having surgery during follow-up period) between the two groups was estimated using exact methods. The primary outcome and binary secondary outcomes were compared between the two groups using logistic regression analyses to estimate the odds ratio (OR) and 95% CI. Time to failure was analysed with Kaplan-Meier survival curves, and Cox regression models to estimate hazard ratios (HRs) and 95% CI. Individuals who had reached endpoint at a previous time point or were permanently lost to follow-up from that time onwards were censored. The number of lashes touching the eye was analysed using zero-inflated Poisson regression to allow for the high proportion of eyes with no lashes touching [27]. The signed-rank test was used to analyse the difference in the number of lashes touching the eye between baseline and 24-mo follow-up. Changes in LogMAR visual acuity score were analysed by logistic regression. A multivariable logistic regression model was used to assess associations of failure with potential explanatory factors. Variables that were associated with the outcome on univariable analyses (*p*<0.2) were retained in the multivariable model. For participants in the surgery arm, the cumulative risk of adverse surgical outcomes was estimated, including recurrence (≥one lashes touching the eye or clinical evidence of epilation), granuloma, and lid notching.

## Results

### Participant Recruitment and Flow

**Figure 1:**
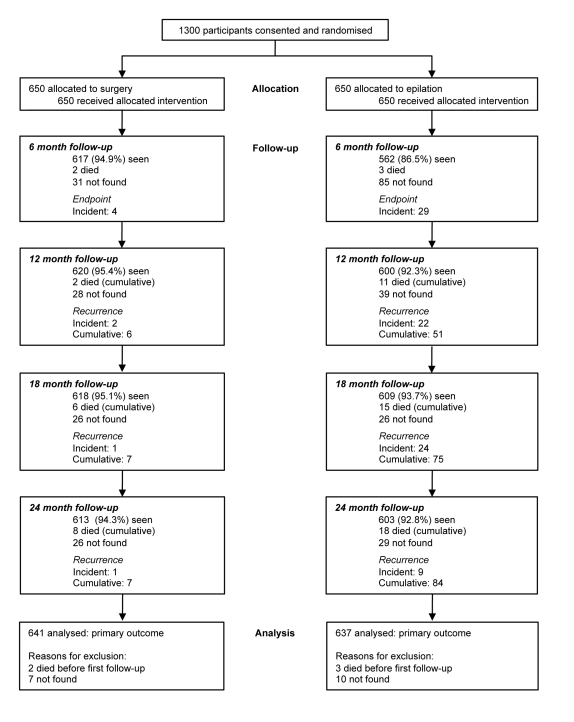
Trial profile.

Trial participants were recruited between March and June 2008. Several thousand people presented with eye complaints during the course of the surgical outreach campaign and were assessed for eligibility for this trial. The majority had other ophthalmic conditions such as cataract. A total of 1,300 consecutive eligible individuals were identified, all of whom consented to participate, were randomised, and received their allocated treatment (650 participants per group) (Figure 1).

Primary outcome data are available for 637/650 (98.0%) participants in the epilation group and 641/650 (98.6%) participants in the surgery group (Figure 1). Overall, 84.1% of participants were seen at all five scheduled visits over 24 mo: 517/650 (79.5%) in the epilation group and 576/650 (88.6%) in the surgery group. There were slightly fewer follow-up examinations in the epilation group (2,374/2,600 [91.3%]) compared with the surgery group (2,467/2,600 [94.9%], *p*<0.001). 22 participants were not seen after baseline (13 epilation group, nine surgery group, *p* = 0.39).

### Baseline Demographic and Clinical Characteristics

All participants were Ethiopians of Amharan ethnicity. Their mean age was 50.3 y and the majority (66.4%) were female. Baseline sociodemographic characteristics were generally balanced by randomisation group, although there were slightly fewer female participants in the epilation group than the surgery group (63.9% versus 68.9%) (Table 1). Clinical features were also balanced (Table 1) with the exception of central corneal opacity (CC2/CC3 or CO), which was more frequent in the epilation group (175; 26.9%) than the surgery group (137; 21.1%) at baseline.

**Table 1:** Baseline demographic and clinical characteristics of participants in each arm of the trial.

Characteristic	Surgery, *n* = 650		Epilation, *n* = 650	
	*n*	Percent or 95% CI	*n*	Percent or 95% CI
***Gender (female)***	448	(68.9%)	415	(63.9%)
***Age (y)***				
18–30	69	(10.6%)	67	(10.3%)
30–39	113	(17.4%)	108	(16.6%)
40–49	156	(24.0%)	150	(23.1%)
50–59	160	(24.6%)	158	(24.3%)
60–69	112	(17.2%)	122	(18.8%)
70+	40	(6.2%)	45	(6.9%)
Mean (SD, 95% CI)	49.9	(14.4, 48.8–51.0)	50.7	(14.5, 49.6–51.8)
***Illiterate***	581	(89.4%)	588	(90.5)
***BMI***, mean (SD, 95% CI)	19.9	(2.3, 19.7–20.1)	20.0	(2.4, 19.8–20.2)
***Study eye (right)*** a	319	(49.1%)	302	(46.5%)
***Best corrected LogMAR VA in study eye***				
−0.2 to 0.3	243	(37.7%)	211	(32.5%)
0.3–0.7	261	(40.5%)	264	(40.7%)
0.7–1.1	83	(12.9%)	89	(13.7%)
1.1–2.0	19	(3.0%)	28	(4.3%)
CF/HM/PL	30	(4.7%)	51	(7.9%)
NPL	9	(1.4%)	6	(0.9%)
Not measurable^b^	5	(0.8%)	1	(0.2%)
***Entropion grade***				
0	276	(42.5%)	292	(44.9%)
1	258	(39.7%)	254	(39.1%)
2	113	(17.4%)	102	(15.7%)
3	3	(0.5%)	2	(0.3%)
4	0	(0.0%)	0	(0.0%)
***Trichiasis (number of lashes touching eye)***				
Mean (95% CI)	1.52	(1.43–1.62)	1.62	(1.52–1.72)
None, epilating	124	(19.1%)	113	(17.4%)
1–5 lashes	526	(80.9%)	537	(82.6%)
***Trichiasis position***				
Corneal ± peripheral	462	(71.1%)	470	(72.3%)
Peripheral only	188	(28.9%)	180	(27.7%)
***Lower lid TT (present)***	70	(10.8%)	57	(8.8%)
***Corneal opacity – field grading***				
CC0, none	345	(53.1%)	330	(50.8%)
CC1, peripheral	168	(25.9%)	145	(22.3%)
CC2a, off centre faint	76	(11.7%)	97	(14.9%)
CC2b, off centre dense	9	(1.4%)	15	(2.3%)
CC2c, central faint	30	(4.6%)	43	(6.6%)
CC2d, central dense	11	(1.7%)	14	(2.2%)
CC3, total central dense	10	(1.5%)	4	(0.6%)
CC4, phthisis	1	(0.2%)	2	(0.3%)
CO or CC2/CC3	137	(21.1%)	175	(26.9%)
***Papillary inflammation***				
None (P0)	52	(8.0%)	66	(10.2%)
Mild (P1)	248	(38.2%)	248	(38.1%)
Moderate (P2)	297	(45.7%)	282	(43.4%)
Severe (P3)	53	(8.1%)	54	(8.3%)
***Conjunctival scarring***				
None (C0)	3	(0.5%)	4	(0.6%)
Mild (C1)	60	(9.2%)	52	(8.0%)
Moderate (C2)	508	(78.2%)	520	(80.0%)
Severe (C3)	79	(12.2%)	74	(11.4%)
***Conjunctivilisation grade***				
0	7	(1.1%)	5	(0.8%)
1	18	(2.8%)	25	(3.9%)
2	165	(25.4%)	146	(22.5%)
3	460	(70.8%)	474	(72.9%)
***Lagophthalmos (present)***	10	(1.5%)	9	(1.4%)
**n ** ***follow-up assessments***				
0	9	(1.4%)	13	(2.0%)
1	10	(1.5%)	13	(2.0%)
2	12	(1.9%)	28	(4.3%)
3	43	(6.6%)	79	(12.2%)
4	576	(88.6%)	517	(79.5%)

a Participants with bilateral TT had one eye randomly selected as the trial eye.

b Unable to cooperate with visual acuity measurement.

BMI, body mass index; CF, count fingers; HM, hand movements; PL, perception of light; NPL, no perception of light; SD, standard deviation; VA, visual acuity.

### Primary Outcome

Overall, 98 individuals developed the primary outcome: 84 (13.2%) in the epilation group and 14 (2.2%) in the surgery group. In the epilation arm 74 had ≥five lashes and an additional ten participants had received repeat surgery elsewhere; in the surgery arm four had ≥five lashes and a further ten had received repeat surgery elsewhere. The difference in cumulative risk of failure was 11.0% (95% CI 8.1%–13.9%). The 95% CI includes the prestated margin of inferiority (Δ, 10.0%), so the trial is inconclusive and does not show evidence of noninferiority of epilation versus surgery, with respect to this prespecified noninferiority margin.

**Figure 2:**
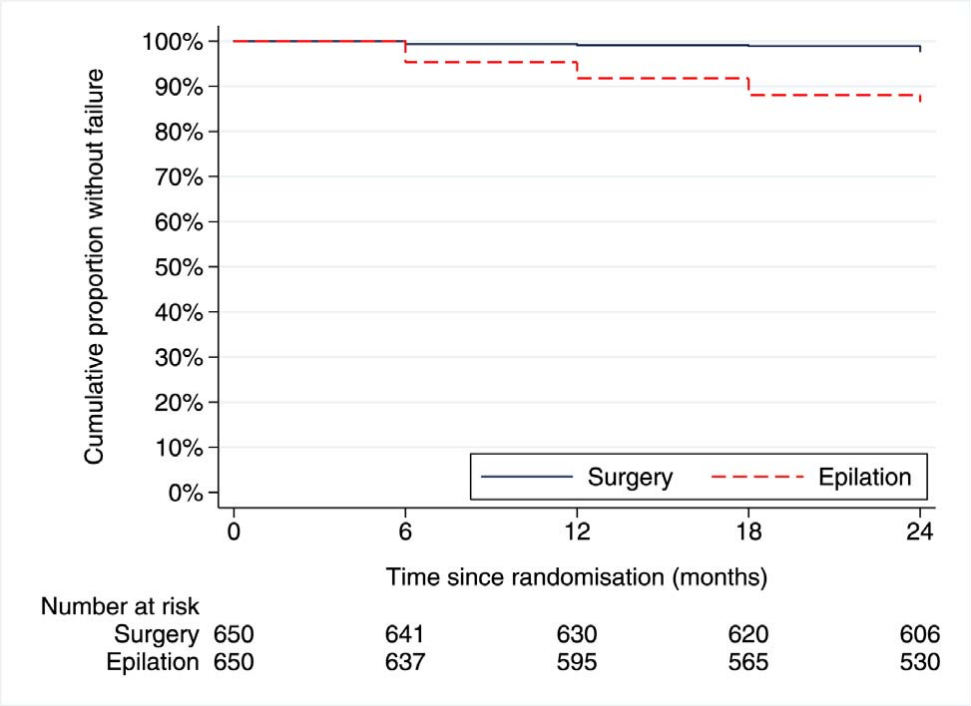
Kaplan Meier graph of time to failure.

The rate of failure was greater in the epilation group (hazard ratio [HR] = 6.38, 95% CI 3.62–11.23) (Figure 2). The mean number of lashes touching the eye at each visit was small (Table 2), but was significantly greater in the epilation group (*p*<0.001). The number of lashes touching the eye reduced significantly between baseline and 24 mo in both groups (*p*<0.001). Among patients in the epilation group, successful epilation (no lashes touching) at baseline was associated with reduced risk of failure (adjusted OR = 0.33, 95% CI 0.13–0.86) and baseline entropion of greater than grade 1 was associated with increased risk (adjusted OR = 1.72, 95% CI 0.98–3.02) (Table 3). There were too few failures in the surgery group to model.

**Table 2:** Clinical characteristics of participants in each arm of the trial at 12 and 24 mo.

Characteristic	12 mo				24 mo			
	Surgery, *n* = 620		Epilation, *n* = 598		Surgery, *n* = 613		Epilation, *n* = 603	
***Trichiasis - mean n lashes touching eye (95% CI)***	0.11	(0.07–0.14)	1.00	(0.89–1.11)	0.09	(0.06–0.12)	0.95	(0.84–1.06)
***Best corrected LogMAR VA in trial eye***								
−0.2 to 0.3	225	(36.4%)	189	(31.8%)	200	(32.7%)	167	(27.8%)
0.3–0.7	261	(42.2%)	231	(38.8%)	272	(44.4%)	258	(43.0%)
0.7–1.1	72	(11.6%)	95	(16.0%)	79	(12.9%)	93	(15.5%)
1.1–2.0	26	(4.2%)	33	(5.6%)	25	(4.1%)	34	(5.7%)
CF/HM/PL	30	(4.9%)	37	(6.2%)	28	(4.6%)	40	(6.7%)
NPL	5	(0.8%)	10	(1.7%)	8	(1.3%)	8	(1.3%)
***LogMAR change (baseline to follow-up)***								
>0.3 worse	49	(8.0%)	66	(11.1%)	71	(11.7%)	87	(14.5%)
0.1–0.3 worse	119	(19.3%)	108	(18.2%)	136	(22.4%)	137	(22.9%)
Within 0.1 (same)	308	(50.0%)	293	(49.3%)	277	(45.6%)	261	(43.6%)
0.1–0.3 better	97	(15.8%)	97	(16.3%)	85	(14.0%)	75	(12.5%)
>0.3 better	43	(7.0%)	30	(5.1%)	38	(6.3%)	39	(6.5%)
***Entropion grade***								
0	598	(96.5%)	223	(37.3%)	588	(95.9%)	167	(27.7%)
1	19	(3.1%)	219	(36.6%)	18	(2.9%)	162	(26.9%)
2	3	(0.5%)	154	(25.8%)	7	(1.1%)	272	(45.1%)
3	0	(0.0%)	2	(0.3%)	0	(0.0%)	2	(0.3%)
4	0	(0.0%)	0	(0.0%)	0	(0.0%)	0	(0.0%)
***Corneal opacity – field grading***								
CC0, none	344	(55.5%)	275	(46.0%)	354	(57.8%)	290	(48.1%)
CC1, peripheral	151	(24.4%)	147	(24.6%)	136	(22.2%)	140	(23.2%)
CC2a, off centre faint	70	(11.3%)	102	(17.1%)	67	(10.9%)	98	(16.3%)
CC2b, off centre dense	14	(2.3%)	18	(3.0%)	15	(2.5%)	25	(4.2%)
CC2c, central faint	22	(3.6%)	35	(5.9%)	18	(2.9%)	28	(4.6%)
CC2d, central dense	12	(1.9%)	16	(2.7%)	15	(2.5%)	14	(2.3%)
CC3, total central dense	6	(1.0%)	2	(0.3%)	7	(1.1%)	5	(0.8%)
CC4, phthisis	1	(0.2%)	3	(0.5%)	1	(0.2%)	3	(0.5%)
***Corneal opacity change, baseline to follow-up – photographic grading***								
More corneal opacity	7	(1.1%)	12	(2.0%)	25	(4.1%)	33	(5.5%)
No change	602	(97.3%)	575	(96.8%)	579	94.6%)	561	(93.3%)
Less corneal opacity	10	(1.6%)	7	(1.2%)	8	(1.3%)	7	(1.2%)
***Corneal opacity change, baseline to follow-up – field grading***								
>3 grades worse	1	(0.2%)	2	(0.3%)	1	(0.2%)	1	(0.2%)
3 grades worse	0	(0.0%)	0	(0.0%)	1	(0.2%)	1	(0.2%)
2 grades worse	17	(2.9%)	27	(4.5%)	18	(2.9%)	22	(3.7%)
1 grade worse	61	(9.8%)	75	(12.5%)	44	(7.2%)	77	(12.8%)
No change	452	(72.9%)	418	(69.9%)	457	(74.6%)	436	(72.3%)
1 grade better	72	(11.6%)	50	(8.4%)	71	(11.6%)	44	(7.3%)
2 grades better	16	(2.6%)	23	(3.9%)	19	(3.1%)	19	(3.2%)
3 grades better	1	(0.2%)	3	(0.5%)	1	(0.2%)	3	(0.5%)
>3 grades better	0	(0.0%)	0	(0.0%)	1	(0.2%)	0	(0.0%)
***Papillary inflammation***								
None (P0)	172	(27.7%)	137	(22.9%)	328	(53.5%)	269	(44.6%)
Mild (P1)	279	(45.0%)	251	(42.0%)	130	(21.2%)	130	(21.6%)
Moderate (P2)	163	(26.3%)	191	(31.9%)	147	(24.0%)	186	(30.9%)
Severe (P3)	6	(1.0%)	19	(3.2%)	8	(1.3%)	18	(3.0%)
***Conjunctivilisation grade***								
0	115	(18.6%)	7	(1.2%)	4	(12.1%)	5	(0.8%)
1	88	(14.2%)	9	(1.5%)	63	(10.3%)	6	(1.0%)
2	258	(41.6%)	83	(13.9%)	309	(50.4%)	54	(9.0%)
3	159	(25.7%)	499	(83.4%)	167	(27.2%)	537	(89.2%)
***Lagophthalmos (present)***	9	(1.45%)	11	(1.8%)	2	(0.3%)	1	(0.2%)

CF, count fingers; HM, hand movements; PL, perception of light; NPL, no perception of light; VA, visual acuity.

**Table 3:** Univariable and multivariable associations for reaching endpoint in the epilation group.

Variable	OR	95% CI	*p*-Value
**Univariable analysis**			
No lashes at baseline (successful epilation)	0.27	0.11–0.68	0.005
Entropion (>grade 1) at baseline	1.75	1.08–2.83	0.02
Conjunctivalisation^a^	1.51	0.97–2.33	0.07
Conjunctival bacteria at baseline	1.00	0.51–1.98	0.99
Persistent inflammation (grade 3+ for 3+ examinations)	1.70	1.08–2.67	0.02
Female	0.96	0.60–1.55	0.88
Age<30 y	2.38	1.28–4.40	0.006
Literate	1.52	0.76–3.05	0.24
BMI<18	1.48	0.86–2.54	0.15
**Multivariable logistic regression model**			
No lashes at baseline (successful epilation)	0.33	0.13–0.86	0.02
Entropion (>grade 1) at baseline	1.72	0.98–3.02	0.06
Conjunctivalisation	1.68	0.94–3.00	0.08
Persistent inflammation (grade 3+ for 3+ examinations)	1.52	0.90–2.57	0.12
Age<30 y	2.00	0.91–4.39	0.09
BMI<18	1.47	0.79–2.75	0.22

a Likelihood ratio test, no evidence of nonlinearity. Therefore combined measure of conjunctivilisation used in logistic regression model.

BMI, body mass index.

### Visual Acuity

There was no evidence of a difference in change in visual acuity (Table 2) from baseline to follow-up between the two groups at either 12 mo (OR = 1.17, 95% CI 0.87–1.59) or 24 mo (OR = 1.16, 95% CI 0.88–1.53). There was similarly no evidence of a difference within randomisation group between the baseline and 24-mo visual acuity (paired *t*-test: surgery group *p* = 0.88; epilation group *p* = 0.99).

### Corneal Opacification

#### Photograph comparison

There was little change in CO, and no significant differences between the two groups. Comparison of the baseline and 24-mo follow-up corneal photographs found incident or progressive corneal opacity in 58 eyes (5.5% epilation; 4.1% surgery; *p* = 0.25).

#### Field grading

Comparison of baseline with follow-up field scores found moderate variation within both groups. The majority of patients in each arm had no change and most differences were of one grade (Table 2). There was a significant difference in change in CO between baseline and 24 mo, with a greater proportion of patients in the epilation arm having progression between baseline and 24 mo (16.8% epilation; 10.4% surgery; *p* = 0.001). However, using the detailed WHO FPC trachoma grading system for the analysis of the field scores there was no statistically significant difference between the two groups in the proportion of eyes with incident or progressive central CO between baseline and 24 mo (6.5% epilation; 4.7% surgery; *p* = 0.19).

### Surgical Outcomes

Trichiasis recurred (defined as one or more lashes touching the eye, or repeat surgery by another provider) in 114 (17.5%) individuals in the surgery group by 24 mo: 103 had minor TT recurrence, five had major TT recurrence, and six had repeat surgery from another provider. For comparison, in the epilation arm at 24 mo 298 (49.4%) had one or more lashes touching the eye. Surgery was associated with a marked reduction in entropion grade at 24 mo (4% versus 72% entropion grade ≥1; OR = 0.02, 95% CI 0.01–0.25) and lid margin conjunctivilisation grade at 24 mo (78% versus 98% conjunctivilisation grade ≥2; OR = 0.05, 95% CI 0.03–0.06) when compared to epilation. Adverse outcomes in the surgery arm are presented in Table 4.

**Table 4:** Postsurgical complications (a) at 7–10-d follow-up and (b) at any subsequent follow-up.

Outcome	*n* Individuals	
**(a) Early postoperative (7–10 d) complications**		
Undercorrection (lashes touching or nearly touching globe)	5	(0.8%)
Overcorrection	2	(0.3%)
Infection/red eye	3	(0.5%)
Infection and undercorrection	1	(0.2%)
Bleeding	3	(0.5%)
**(b) Complications during 2-y follow-up**		
Recurrence	105	(16.2%)
Granuloma	18	(2.9%)
Notching	29	(4.7%)

### Symptoms

Participants were asked about their symptoms (Table 5). At 12 mo postrandomisation, participants in the surgery arm recalled greater treatment pain than those in the epilation group (*p*<0.001) but reported better subjective improvement in vision (*p*<0.001) and less eye-watering (*p*<0.001).

**Table 5:** Symptoms reported by participants at 12- and 24-mo follow-ups.

Characteristic	12 mo				24 mo			
	Surgery, *n* = 620		Epilation, *n* = 598		Surgery, *n* = 613		Epilation, *n* = 603	
Vision								
Better	484	(78.2%)	200	(33.4%)	545	(88.9%)	153	(25.4%)
Same	87	(14.0%)	297	(49.7%)	59	(9.6%)	446	(74.1%)
Worse	48	(7.8%)	101	(16.9%)	9	(1.5%)	3	(0.5%)
Eye pain	272	(44.0%)	417	(69.7%)	353	(57.6%)	498	(82.6%)
Eye watering	318	(51.4%)	378	(63.2%)	366	(59.7%)	423	(70.2%)
Dry eye	138	(22.3%)	162	(27.1%)	134	(21.9%)	167	(27.7%)
Treatment pain^a^								
None	49	(8.0%)	167	(33.3%)	—		—	
Mild	274	(44.8%)	193	(38.5%)	—		—	
Moderate	17	(2.8%)	6	(1.2%)	—		—	
Severe	262	(42.8%)	136	(27.1%)	—		—	
Don't remember	10	(1.6%)	0	(0.0%)	—		—	

a As recalled at 12 mo.

### Management at 24 Months

At 24 mo postrandomisation, 593/603 individuals in the epilation group had not had surgical treatment of their trichiasis. When offered free surgery, 185/593 (31%) accepted.

## Discussion

We found no evidence that epilation is noninferior to surgery for the management of minor trichiasis, using a noninferiority margin of 10% for the difference in cumulative risk of failure. Therefore, in statistical terms, the trial is inconclusive. The proportion failing was 13.2% in the epilation group and 2.2% in the surgical group (difference = 11.0%; 95% CI 8.1%–13.8%). A relatively small proportion of patients in the epilation arm had evidence of progressive trichiasis; the rate of progression was slower than those previously reported [4],[21]. Failure in the epilation arm was less frequent when the subject had been successfully epilating prior to enrolment and was more frequent if moderate entropion was present.

Overall, there was no significant difference in change in visual acuity between the groups. However, following surgery more people subjectively felt their vision had improved compared to those in the epilation group. This difference may have been due to watering of the eye (epiphora), which was reported more frequently by the epilation group, or disturbance caused by residual lashes. However, there could have also been an element of reporter bias, as participants who have received surgery may have felt more obliged to report an improvement to the team that provided the surgery. There was no significant change in vision over 2 y within either group. Two previous studies have reported a modest short-term improvement in visual acuity following surgery; however, both studies included many patients with more severe trichiasis; these people probably have greater potential for visual improvement [3],[28].

Direct comparison of the baseline with 1- and 2-y corneal photographs found that there was very little change in corneal appearance and that there was no difference between the groups. The comparison of the longitudinal field grading scores suggested more variation occurred in both groups between time points, with slightly more progression in the epilation arm compared to the surgery arm. When the field scores were analysed using the WHO FPC grading system, there was no significant difference in progression between the groups. Our results are consistent with two previous longitudinal nonrandomised studies that examined corneal change in individuals with minor trichiasis, which found no difference in the development of new CO between those accepting surgery and those declining it (who were mostly epilating) [4],[18],[21].

Overall, the authors consider that direct comparison of high-resolution digital photographs viewed at high magnification offers the most reliable measure of change in corneal appearance. Whilst it is possible that photographs are slightly less sensitive than careful direct clinical examination for very faint haze (which would not normally be considered trachomatous CO), it is unlikely that visually significant lesions would be missed. The kappa scores for the comparison of the field and photographic grading indicated good agreement between the observers. However, when a very detailed grading system such as the one developed for this study is used, some changes will lie at the boundary between two grades; even when all observations are performed by the same experienced ophthalmologist it is inevitable that some (unchanged) lesions will be graded slightly differently on different occasions, which we think accounts for much of the change reported by field scores. Of note, the two groups were not balanced at baseline for the amount of central corneal opacity (CO or CC2/CC3); by chance there was significantly more CO in those randomised to the epilation group, which may have led to more “observation noise” occurring in this group. This “observation noise” can be overcome by direct comparison of photographs, and explains why overall much less change was observed between the photographs. Photographs also offer the opportunity to detect change in lesions that may not result in a change in the categorical grade, so in this respect they are more sensitive than the categorical field grading.

Surgery, when there is no recurrence, offers a potentially long-lasting solution, whilst epilation is an ongoing intervention, which needs to be repeated when trichiatic lashes regrow. Therefore, it is to be expected that surgery would be better at preventing any eyelashes from touching the eye. However, surgery is not without problems. Although few individuals in the surgery arm reached endpoint, 16% developed recurrent TT. This number is slightly lower than that generally reported in trials and case series and is probably due to the relatively mild stage of the disease in the people recruited compared to other studies and the extra training and high surgical volume of the surgeons participating in this study [3],[18]–[20]. Lid margin notching, which is cosmetically unsatisfactory, was found in 5% of participants in the surgery arm and conjunctival granulomas requiring an additional minor procedure in 3%.

There are several considerations in interpreting the results of this trial. The choice of Δ in noninferiority trials is inevitably open to debate. In selecting a noninferiority margin of 10%, we took a relatively conservative view as to what would be programmatically acceptable, given the secondary benefits of acceptability and availability of epilation. If one were to only consider the clinical outcome in evaluating whether there is a place for epilation in the management of mild trichiasis, one would reject it, as the data from this study indicate that surgery is better at preventing lashes touching the eye. However, it should be borne in mind that the surgery in this trial was performed by surgeons selected for the high quality of their operating ability; they received refresher training and were routinely operating large numbers of cases. It is likely that the results they achieved were better than those that might be obtained under standard conditions. Therefore, under operational conditions the difference in clinical outcome between surgery and epilation would probably be smaller.

Moreover, the reality on the ground is that a large majority of the approximately 8 million people who have TT are not receiving surgical treatment. Given the enormous backlog of untreated cases and slow progress in addressing this, surgery is unlikely to be available to very large numbers of people living with trichiasis for at least the medium term. This situation is due to multiple challenges including insufficient numbers of health workers trained in TT surgery, who generally have conflicting responsibilities, limited resources, and barriers to accessing surgery such as low awareness of services and nonfinancial costs such as the time off work recuperating [10],[11]. Some of these challenges can and will be overcome with improved service organisation and investment. However, despite huge efforts to scale up trichiasis surgery services, for many patients the surgical services are likely to remain unavailable, inaccessible, or prohibitively expensive for the medium term [7],[12]–[14]. Furthermore, many patients, particularly those with mild disease, decline the offer of surgery, even where this is being provided free of charge at community level, preferring to epilate [7],[13]–[15]. Of note at the close of this trial all individuals in the epilation group were offered free, community-based surgery; only 31% accepted. Epilation is cheap, can be provided immediately during the first encounter of the patient with the service, delivered by volunteers with minimal training at community level to large numbers of people, and is generally acceptable to patients as a treatment modality.

Whether there is a role for epilation in trachoma control is ultimately a decision for individual national programmes, which would need to take into account a number of locally specific factors including the availability of staff and resources to provide a high quality surgical service and the acceptability of surgery to the population they serve.

There are several limitations to this study. Differential loss to follow-up is a potential source of bias in noninferiority trials, as one arm might have fewer opportunities to demonstrate failure. There were slightly fewer follow-up assessments in the epilation arm; however, this difference was not great and would be unlikely to have materially affected the trial outcome. There was a very slight difference between the two arms of the trial in the proportion that are female and, as discussed above, in the baseline prevalence of CO. These slight differences occurred by chance, as there were no errors in randomisation allocation. Detecting differences in change in both corneal disease and visual acuity may also require a longer follow-up period than the 2 y in this study.

Overall, the data from this trial and earlier nonrandomised studies suggest that, for people with minor trichiasis, repeated epilation is associated with visual acuity and corneal change outcomes that are comparable to surgery over a 2-y period [4],[18],[21]. Where surgery is available and patients are willing to accept it, surgery should be performed, as the disease tends to progress, albeit quite slowly. However, we suggest that for individuals with minor trichiasis (who represent about half of all those with TT) epilation should be considered where surgery is either not available or declined by the patient.

## Supporting Information

Table S1 **(a) Expanded grading system for entropion, conjunctivilisation, and CO. (b) Conjunctivilisation: anterioplacement of the muco-cutaneous junction of the upper eyelid. (c) Corneal scarring (assessed with eye in primary position).**
(DOC)

Text S1 **Trial protocol.**
(DOC)

Text S2 **CONSORT checklist.**
(DOC)

## Abbreviations

CC: central corneal scarring
CO: corneal opacification
OR: odds ratio
TT: trachomatous trichiasis
WHO: World Health Organization

## References

[1] Mariotti SP, Pascolini D, Rose-Nussbaumer J (2009). Trachoma: global magnitude of a preventable cause of blindness.. Br J Ophthalmol.

[2] West ES, Munoz B, Imeru A, Alemayehu W, Melese M (2006). The association between epilation and corneal opacity among eyes with trachomatous trichiasis.. Br J Ophthalmol.

[3] Burton MJ, Kinteh F, Jallow O, Sillah A, Bah M (2005). A randomised controlled trial of azithromycin following surgery for trachomatous trichiasis in the Gambia.. Br J Ophthalmol.

[4] Burton MJ, Bowman RJ, Faal H, Aryee EA, Ikumapayi UN (2006). The long-term natural history of trachomatous trichiasis in the Gambia.. Invest Ophthalmol Vis Sci.

[5] Reacher MH, Munoz B, Alghassany A, Daar AS, Elbualy M (1992). A controlled trial of surgery for trachomatous trichiasis of the upper lid.. Arch Ophthalmol.

[6] World Health O (2006). Trachoma control - a guide for programme managers.

[7] Bowman RJ, Faal H, Jatta B, Myatt M, Foster A (2002). Longitudinal study of trachomatous trichiasis in The Gambia: barriers to acceptance of surgery.. Invest Ophthalmol Vis Sci.

[8] World Health Organization (2004). Report of the 2nd global scientific meeting on trachoma.

[9] Berhane Y, Worku A, Bejiga A (2006). National survey on blindness, low vision and trachoma in Ethiopia.

[10] Lewallen S, Mahande M, Tharaney M, Katala S, Courtright P (2007). Surgery for trachomatous trichiasis: findings from a survey of trichiasis surgeons in Tanzania.. Br J Ophthalmol.

[11] Habtamu E, Rajak SN, Gebre T, Zerihun M, Genet A (2011). Clearing the backlog: trichiasis surgeon retention and productivity in northern Ethiopia.. PLoS Negl Trop Dis.

[12] Courtright P (1994). Acceptance of surgery for trichiasis among rural Malawian women.. East Afr Med J.

[13] Oliva MS, Munoz B, Lynch M, Mkocha H, West SK (1997). Evaluation of barriers to surgical compliance in the treatment of trichiasis.. Int Ophthalmol.

[14] Mahande M, Tharaney M, Kirumbi E, Ngirawamungu E, Geneau R (2007). Uptake of trichiasis surgical services in Tanzania through two village-based approaches.. Br J Ophthalmol.

[15] Bowman RJ, Soma OS, Alexander N, Milligan P, Rowley J (2000). Should trichiasis surgery be offered in the village? A community randomised trial of village vs. health centre-based surgery.. Trop Med Int Health.

[16] Reacher M, Foster A, Huber J (1993). Trichiasis surgery for trachoma: the bilamellar tarsal rotation procedure.

[17] World Health Organization (2005). Final assessment of trichiasis surgeons.

[18] Burton MJ, Bowman RJ, Faal H, Aryee EA, Ikumapayi UN (2005). Long term outcome of trichiasis surgery in the Gambia.. Br J Ophthalmol.

[19] Khandekar R, Mohammed AJ, Courtright P (2001). Recurrence of trichiasis: a long-term follow-up study in the Sultanate of Oman.. Ophthalmic Epidemiol.

[20] West ES, Mkocha H, Munoz B, Mabey D, Foster A (2005). Risk factors for postsurgical trichiasis recurrence in a trachoma-endemic area.. Invest Ophthalmol Vis Sci.

[21] Bowman RJ, Faal H, Myatt M, Adegbola R, Foster A (2002). Longitudinal study of trachomatous trichiasis in the Gambia.. Br J Ophthalmol.

[22] Melese M, West ES, Alemayehu W, Munoz B, Worku A (2005). Characteristics of trichiasis patients presenting for surgery in rural Ethiopia.. Br J Ophthalmol.

[23] Rajak SN, Habtamu E, Weiss HA, Bedri A, Gebre T (2011). Epilation for trachomatous trichiasis and the risk of corneal opacification.. Ophthalmology.

[24] Dawson CR, Jones BR, Tarizzo ML (1981). Guide to trachoma control.

[25] Thylefors B, Dawson CR, Jones BR, West SK, Taylor HR (1987). A simple system for the assessment of trachoma and its complications.. Bull World Health Organ.

[26] West SK, West ES, Alemayehu W, Melese M, Munoz B (2006). Single-dose azithromycin prevents trichiasis recurrence following surgery: randomized trial in Ethiopia.. Arch Ophthalmol.

[27] Lee AH, Wang K, Scott JA, Yau KK, McLachlan GJ (2006). Multi-level zero-inflated poisson regression modelling of correlated count data with excess zeros.. Stat Methods Med Res.

[28] Woreta TA, Munoz BE, Gower EW, Alemayehu W, West SK (2009). Effect of trichiasis surgery on visual acuity outcomes in Ethiopia.. Arch Ophthalmol.

