# Supplementary material for: Surgery Versus Epilation for the Treatment of Minor Trichiasis in Ethiopia: A Randomised Controlled Noninferiority Trial
Source: PLoS Med. 2011 Dec 13;8(12):e1001136. doi: 10.1371/journal.pmed.1001136 (PMC3236738; doi:10.1371/journal.pmed.1001136)

**Strategies for the management of trachomatous trichiasis**

**Trial 2: Randomised controlled trial of epilation against surgery for management of minor trachomatous trichiasis**

**Study Protocol**

**Summary**

Trachoma is the leading infectious cause of blindness worldwide. Chronic keratoconjunctivitis caused by Chlamydia trachomatis causes scarring of the inner eyelid. This in turn causes in-turning of the eyelid (entropion), causing the eyelashes to turn inwards (trichiasis) and rub the cornea, resulting in scarring and visual impediment. SAFE is the WHO initiative for the control of trachoma. It comprises, Surgery, Antibiotics, Facial cleanliness and Environmental improvement. The surgery aims to correct the trichiasis, thereby preventing further visual loss. However surgery can have a high recurrence rate. It is unknown whether surgery should be carried out on all degrees of severity of trachomatous trichiasis or whether epilation should be practised in less severe case. This trial aims to determine if epilation is non-inferior for minor (5 or less eyelashes touching the globe) trichiasis.

**Trial Location and format**

The trials are planned to be undertaken in the West Gojjom zone, Amhara region.

Trachomatous trichiasis surgical campaigns will be organised in several health centres and/or health posts. Recruitment will take place during these campaigns.

**A: Trial Recruitment Phase (see diagram 1 below)**

1. **Identify locations (see diagram 2 below) for different parts of recruitment process**

Team Members who will perform this (appendix A)

SR (research fellow) and nurse 1 (examination nurse)

1. **Presentation to patients and accompanying people**

Team Members

Assistants 1 and 2 (consent field workers)

Procedure

- 1. Hand out project information sheets.
  2. Introduction re who we are.
  3. Info about what is known about trachoma.
  4. Info about what is not known about trachoma.
  5. Info about the trials.
  6. Info about safety to patient.
  7. Info about what we will do from now.

Late comers will receive similar presentation individually or in small groups.

1. **Triage Patient**

Team members

Field worker to call SR or EM to triage Patient to:

- 1. Trial 1 or trial 2
  2. Treatment for right eye/left eye/bilateral
  3. Decide which is trial eye if bilateral TT (using random eye table)

Procedure

1. Patients with trachomatous eye disease and no exclusion criteria, enter into study.
2. Patients with trachomatous eye disease and exclusion criteria. Do not enter into study, but give standard trachoma treatment, including surgery if required.
3. Patients with non-trachomatous eye disease:
   - 1. Emergency: to be seen by ophthalmologist or ophthalmological nurse and treated if necessary or arrangements made for urgent referral to local eye facility
     2. Non-emergency: to be seen by ophthalmological nurse and instructions for attendance at local eye unit with referral letter if required.

Inclusion criteria – trial 1

Patients with major trachomatous trichiasis (greater than 5 eyelashes touching the globe of either or both eyes), who consent to be part of the trial and do not have any of the exclusion criteria below.

Inclusion criteria – trial 2

Patients with minor trachomatous trichiasis (5 or less eyelashes touching the globe of either or both eyes), who consent to be part of the trial and do not have any of the exclusion criteria below.

Exclusion criteria for both trials

- Age less than 18 years
- Previous eyelid surgery
- Medically unfit, BP systolic >170, diastolic >110.
- Pregnant

1. **Consent**

Team Members

Assistants 1 and 2 (Consent field workers)

Procedure

- 1. Check if they heard initial lecture and if so whether they understood it.
  2. If not, then explain trachoma/trial.
  3. Check if they have received the information leaflet and had time to read it. If not received then give this to patient and time to read.
  4. If patient illiterate, information to be read to them by trained assistant.

Procedure to follow if patient does not consent or is not competent to consent

- Becomes non-trial patient.
- Continue as per local protocol, ensuring that patient is still offered surgery by the project team.

Identification of which trial each patient is in

Attempt to sit patients being recruited into the two different trials in different waiting areas. Give patient large piece of card with trial number and eye to be treated (right/left/bilateral) written on it.

1. **Questionnaire (see appendix B)**

Team Members

Assistants 1 and 2 (Consent field workers)

Contents

Part 1: demographics

Part 2 (part 3 of the study record form, appendix B): symptoms, surgical and epilation history

1. **Visual acuity, height and weight**

Team Members

Assistants 3 and 4 (Vital statistics field workers)

Procedure

1. Logmar 4m tumbling E chart
2. Height
3. Weight
4. **Examine and photograph patient**

Team Members

SR, assistant 5 (examination field worker) and field co-ordinator when triaging finished

Procedure

See examination protocol, appendix B

1. **Randomise patient to one of the two arms of appropriate trial for that patient (appendix C)**

Team Members

Assistant 6 (randomisation field worker)

Procedure

The full randomisation procedure is described in appendix C.

The part of this describing the ‘in field’ procedure is described below. This will be performed by a dedicated randomisation field worker.

1. Randomisation field worker will be working in location not visible to other project team members.
2. After consent and clinical examination the patient is sat in a waiting area.
3. When a surgeon is available for operating, the surgeon finds the randomisation field worker and together they take an envelope from that surgeon’s pile. The will open the envelope and record the instruction on masterlist E2 (see below). This will be recorded on two copies of masterlist two, with one copy being kept by the randomisation field worker and the second copy being given to a person independent of the trial. A new masterlist E2 will be used on each trial day at the end of every day.
4. If the instruction in the envelope is for surgery the randomisation field worker and the surgeon take the patient through to the theatre.
5. Field worker will:
   1. Take 3 (if unilateral surgery required) or 6 (if bilateral) silk sutures from the suture store and give to the surgeon. They will ask the surgeon to double check that this is the correct (silk) suture.
   2. They will use masterlist E3 (see below) to record whether the patient needs suture removal to be performed in 7-10 days. This will be recorded on two copies of masterlist E3.

Master list E2

| Date | Study Number | Name | Randomisation Envelope | Study Eye | Treatment given |
| --- | --- | --- | --- | --- | --- |
|  | 1 | XX | E1/0001 |  | E |
|  | 2 | Xx | E2/0001 |  | S |
|  | 3 | Xx | E3/0001 |  | E |
|  | 4 | Xx | E1/0002 |  | E |
|  | 5 | Xx | E3/0002 |  | S |
|  | 6 | Xx | E2/0002 |  | S |

Masterlist E3 – suture removal

| Unique identifier | Name | Date of surgery | Date for suture removal | Eye for suture removal (R/L/B) | Sutures successfully removed  (yes or no) |
| --- | --- | --- | --- | --- | --- |
|  |  |  |  |  |  |
|  |  |  |  |  |  |
|  |  |  |  |  |  |

1. **(a) Surgery**

Team Members

Nurses 2, 3 and 4 (surgical nurses), assistant 7 (surgical assistant). Up to five different surgical nurses will be involved in operating patients in the trial, although unlikely to be more than three nurses working on the same day.

Procedure

- 1. Nurse 2, 3 or 4 to operate on patient
  2. Nurse 2, 3 or 4 will record the surgical outcome on masterlist E4 (see below)
  3. Nurse 2, 3 or 4 to give post-op instructions
     1. Tetracycline
     2. Keep eye clean
     3. Not to rub eye
     4. Return 7-10 days for removal of silk (BLACK) sutures and recording of immediate post-op complications

The Trabut surgical technique will be used (appendix D)

Sterilization will be as per The Trichiasis Surgery for Trachoma WHO manual (appendix E)

All sharps (needles, blades etc) will be disposed of in specialised ‘sharps bins’

Masterlist E4

| Unique identifier | Name | Date of surgery | Surgery routine or complicated | Complication (if applicable) |
| --- | --- | --- | --- | --- |
|  |  |  |  |  |
|  |  |  |  |  |
|  |  |  |  |  |

Procedure to follow if intra-operative complication

- Follow local policy
- However, ensure that same suture as was initially randomised is still used, even if more or less than usual are needed.
- Masterlist S4 (see above) will be used to record any intra-operative complications.

**(b) Epilation training**

Team Members

Assistant 8 (epilation field worker)

Procedure

*A. If patient and suitable relative both present at surgical camp*

1. Take patient and relative to designated epilation training area.
2. Re-explain purpose of trial
3. Explain need for:
   1. Good quality epilation
   2. Regularity of epilation
4. Test near vision of relative.
5. Show forceps to relative and ask if they have epilated previously.
6. Warn of hazards of forceps:
   1. Corneal trauma
   2. Breaking lashes to leave abrasive stubs
   3. Remind that there is a contact number on the information sheet if difficulties do arise and give named person to contact in case of concerns.
7. Tell relative of need for good lighting and undisturbed location.
8. Observe relative epilating.
9. Check lid after epilation:
   1. If epilation fully successful (no trichiatic lashes remain): give epilation pack as per (10)
   2. If epilation partially successful (no lashes touching globe/cornea, but trichiatic lashes remain) (i.e. give advice.
   3. If epilation failed (lashes touching globe/cornea):
      1. Either: repeat full epilation training
      2. Or: ask if another more appropriate relative is available

But do NOT exclude from trial.

1. Give epilation pack:
   1. Forceps
   2. Lid diagram/diary cards in A4 plastic wallet.
   3. Pencil

*B. If patient not with suitable relative*

1. Investigate if suitable relative can be found: consider:
   1. Either: Deferring epilation training until later in the week
   2. Or: project driver to fetch relative.

**B: Trial Follow-up Phase**

1. **7-10 day follow-up for surgical patients**

Team members

Nurse

Driver 2, if patient fails to attend for follow up

1. Find appropriate patients according to suture removal lists from each trial.
2. Record post operative examination on new form for patient
3. Double check that this patient is from trial 2 and therefore has silk (BLACK) sutures.
4. Remove all sutures.
5. Record removal of sutures on masterlist E5
6. Give patient further instructions

Masterlist E5

| Unique identifier | Name | Date of suture removal | Eye of suture removal | Early post operative findings |
| --- | --- | --- | --- | --- |
|  |  |  |  |  |
|  |  |  |  |  |
|  |  |  |  |  |

Examination

1. Are/is there:
   1. Discharge
      1. Purulent
      2. Non-purulent
   2. Obvious granuloma (do NOT evert lid)
   3. Bleeding
   4. Early recurrence
      1. Count eyelashes and record
   5. Skin infection / cellulitis
2. Is further surgery being planned?
   1. When?
   2. Where?

Procedure if early recurrence is identified

Either:

1. Operate immediately, if suitably trained surgeon is present
2. Refer to Bahir Dar for urgent re-op
3. Refer to another local surgical campaign, if there is capacity and a suitably trained surgeon.

Failure to present at 7-10 follow up

1. Is this due to:
   1. Death
   2. Travelled
   3. Unknown
2. Driver and suture removal nurse to take all possible steps to find patient or arrange for their presentation as soon as possible for suture removal.

Late complications

If patient re-presents between the 7-10 day follow up and the 3 month follow up, record reason and presence/absence of any of the above complications and follow procedure above.

1. **6 month follow up**

Team

Field Co-ordinator with SR assistance

Driver 1

Assistants

Aims

Full examination and photography

Swabs

1. **One year follow up**

Team

SR

Field co-ordinator

Assistants

Driver 1

Aims

Full examination and photography

Questionnaire

Visual acuity

Swabs

1. **18 month follow up**

Team

Field co-ordinator with SR assistance

Driver 1

Assistants

Aims

Full examination and photography

Swabs

1. **Two year follow up**

Team

SR

Field co-ordinator

Assistants

Driver 1

Aims

Full examination and photography

Swabs

**Typical project day protocol (diagram 1)**

Triage patient

Identify suitable areas for different parts of recruitment process

Lecture and info sheet to all patients

Not trachoma

Trachoma

Emergency

Non-emergency

Arrangements for urgent attendance at local eye unit or acute treatment in field if required and possible.

Instructions for attendance at local eye unit and referral letter if required

Not suitable for study, i.e. exclusion criteria

Suitable for study 1

Allocate unique patient identifier

Suitable for study 2

Allocate unique patient identifier

Treat in field as per clinical need and patient choice, including surgery if required

Consent

Consent

Questionnaire

Questionnaire

Measure VA, height, weight

Measure VA, height, weight

Examine patient

Examine patient

Photograph

Photograph

Randomise

Randomise

Surgery

Surgery

Epilation training

Post-op instructions

Post-op instructions

Post-op instructions

Nurse 1, assistants 1,2 + SR

Nurse 1

SR/EO/Nurse 1,2,3 or 4

Nurse 1,2,3 or 4

Nurse 2,3 or 4

Assistants 1+2

SR, Nurse 1, Assistnant 5

Assistant 6

Nurse 2,3,4 assistant 7

Assistant 8

Assistant 6

Nurse 2,3,4 assistant 7

SR

Nurses 2,3,4

Nurses 2,3,4

Assistants 3+4

Nurses 2,3,4

**Appendix A – The project team**

**The team**

SR (Research Fellow)

Co-ordination

Examination and clinical photography

Field Co-ordinator (Nurse 1)

Trained in:

1. Examination and will then conduct 6 and 18 month follow up.
2. Triage

Nurse 2, 3 and 4 (surgery nurses)

Trachoma surgeons. Technique validated and standardised by Dr Bedri/Dr Abdul

Nurse 5

Suture removal.

Assistants 1 and 2 (consent field workers)

Trained in:

1. Project explanation/presentation
2. Consent taking
3. Questionnaire administration

Assistants 3 and 4 (Vital statistics field workers)

Trained in measuring

1. Logmar visual acuity
2. Height
3. Weight

Assistant 5 (examination field worker)

Trained in assisting SR with:

1. Examination
2. Photography
3. Microbiological swabbing

Assistant 6 (randomisation field worker)

Trained in how randomisation/masking etc

Assistant 7 (surgical assistant)

Trained in assisting nurses with surgery

Assistant 8 (epilation field worker)

Trained in teaching epilation

Driver 1

Project team driver

Driver 2

Project team driver; suture removal team driver

**Appendix B – Questionnaire and examination protocol and study record form**

**Patient Examination**

1. **General Examination**
   1. **Height in centimetres**
   2. **Weight in kilograms**
   3. **Determine trial eye**

Use pre-designed random eye table

- 1. **Record eye being treated**

1. **Ophthalmic Examination**
   1. **Visual Acuity:**  **EDTRS tumbling E logmar chart at 4m**

a) Best corrected

b) Pinhole vision

To calculate LogMAR visual acuity for 4m:

1. Test to lowest line on which any letter can be read. Complete whole line, even if many mistakes.

2. Take the LogMAR score for the lowest complete line read

3. Add 0.02 x n to this score, where n = the number of letters read from the non-completed line.

N.B

If chart is read at 2m: add 0.3 correction

If chart is read at 1m: add 0.6 correction

**3.2-3.8 Assess Trichiasis**

Assess eyelash position with eye in primary position (looking straight ahead)

- 1. Number of lashes whose point touches globe medial to cornea
  2. Number of lashes whose point touches globe lateral to cornea
  3. Number of lashes whose point touches cornea
  4. Determine grade of trichiasis

| **Trichiasis Grade** | **Definition** |
| --- | --- |
| T 0 | No trichiasis |
| T 1 | Lashes deviated towards the eye, but not touching the globe |
| T 2 | Lashes touching the globe but not rubbing the cornea. |
| T 3 | Lashes constantly rubbing the cornea. |

- 1. Count number of metaplastic lashes right and left eye (*i.e. trichiasis not secondary to entropion*)
  2. Count number of misdirected lashes right and left eye

| Number of metaplastic/misdirected lashes | Right eye | Left eye |
| --- | --- | --- |

- 1. **Count lower lid trichiatic lashes**
  2. **Assess entropion**

1. Assess orientation of the lid margin of the eye in the primary position.

- If necessary, gently raise any excess folds of upper lid skin, *without*, disturbing the position of the upper lid.

*If there is a mixed picture, classify as the worse grade.*

| **Degree of severity** | **Area of entropion** | | |
| --- | --- | --- | --- |
|  | **<50% of lid margin** | **>50% of lid margin** | |
| None | E0 (none) | | |
| Without corneal-lash base contact | E1 (mild) | E2 (moderate) | |
| With corneal-lash base contact | E3 (severe) | | E4 (total) |

None ‘Normal’ lid margin (see glossary) visible

Without corneal-lash base contact Definite inwards rotation of the lid margin, without any lash bases touching the cornea

With corneal-lash base contact Inward rotation of lid margin, with some or all of the lash bases touch the cornea.

**3.10. - 3.12 Assess epilation**

- 1. Is there clinical evidence of epilation? No/yes
  2. How much epilation is there? None, <1/3^rd^, 1/3rd-2/3^rd^, >2/3^rd^
  3. How successful is the epilation? none, successful, <6 lashes, 6+ lashes
  4. **Photograph trichiasis (right and left)**
  5. **Assess plica semilunaris:**

Is the plica semilunaris:

a) Normal: see glossary.

b) Diseased but present. A fold of skin is present, but this is either distorted in shape or scarred down to the underlying conjunctiva.

c) Effaced: the plica semilunaris is absent.

- 1. **Assess for symblepharon (upper or lower): no/yes**

**3.16. – 3.17. Assess for ocular discharge**

- 1. Is there discharge? No/Serous/Purulent/Other (describe)?
  2. **Examine corneal scarring**

If there are more than one corneal scars grade as for worst/most central scar.

a) Grade corneal scarring (see card for diagrammatic representations of corneal scar grades. See appendix F)

C1 Opacity not entering central 4mm

C2a Opacity within central 4mm but not entering within the central 1mm of the cornea. The pupil margin is visible through the opacity.

C2b Opacity within central 4mm but not entering within the central 1mm of the cornea. The pupil margin is not visible through the opacity.

C2c Opacity within central 4mm and entering the central 1mm of the cornea. The pupil margin is visible through the opacity.

C2d Opacity within central 4mm and entering within the central 1mm of the cornea. The pupil margin is not visible through the opacity.

C3 Opacity large enough and dense enough to make whole pupil margin invisible.

C4 Phthisis

- 1. **Photograph cornea**

Photo grading:

1) Size/area

2) Density

i) Mild haziness = mildly obscures iris architecture

ii) Moderate opacity = moderately obscures iris architecture

iii) Severe opacity = iris architecture not visible

If there are >1 opacities, then each should be photo-graded independently.

**Perform bacteriology swab**

- 1. Insert one drop of proxymetacaine
  2. Sweep four times horizontally in the inferior fornix particularly in areas of discharge.
  3. Break swab off into tube containing Stuarts media and seal tube
  4. Label tube
  5. Check labelling done and correct
  6. Record that swab has been performed on the patient record form
  7. Place tube in sample box in the cool box
  8. **Lagophthalmos: no/yes**

**Evert eyelid and continue examination sequence:**

**1) Evert with stick end of swab**

**2) Grade**

**3) Photo**

**4) Swab**

- 1. **Assess the presence of conjunctivalisation of the lid margin (assess with upper lid everted)**

CM 0 No conjunctivalisation of the lid margin

CM 1 The muco-cutaneous junction is located anterior to its normal position, but the whole line is still posterior to the line of Meibomian gland orifices.

CM 2 The muco-cutaneous junction is located anterior to the line of the Meibomian gland orifices for less than 50% of the lid.

CM 3 The muco-cutaneous junction is located anterior to the line of the Meibomian gland orifices for greater than 50% of the lid.

- 1. **Examine upper lid follicles**

a ) Determine grade of follicular reaction – examine only zones 2 and 3, ignore follicles in zone 1

F 0 No follicles.
F 1 1-4 follicles.
F 2 5-10 follicles.
F 3 >10 follicles.

- 1. **Examine upper lid papillary hypertrophy**

1. Determine the grade of severity of papillary hypertrophy:

P 0 Absent: normal appearance

P1 Minimal: individual vascular tufts (papillae) prominent, but deep subconjunctival vessels on the tarsus are not obscured.

P2 Moderate: more prominent papillae and normal vessels appear hazy, even when seen by the naked eye.

P3 Pronounced: conjunctiva thickened and opaque, normal vessels on the tarsus are hidden over more than half of the surface.

b) Determine whether papillary reaction is over less than or greater than 50% of the tarsal area.

Papillary inflammation grading

|  | <50% of tarsal area inflamed | >50% of tarsal area inflamed |
| --- | --- | --- |
| None | 0 | 0 |
| Mild | 1 | 2 |
| Moderate | 3 | 4 |
| Severe | 5 | 6 |

To convert to traditional papillary grading system use this table:

| Degree of inflammation /’redness’ | <50% of tarsal area inflamed | >50% of tarsal area inflamed |
| --- | --- | --- |
| None | P0 | P0 |
| Mild | P1 | P1 |
| Moderate | P2 | P2 |
| Severe | P2 | P3 |

**3.24. – 3.25. Conjunctival scarring**

- 1. Conjunctival scarring if no previous surgery Examine upper lid conjunctival scarring (use remainder of swab stick to evert lid)

C0 No scarring on the conjunctiva

C1 Mild: fine scattered scars on the upper tarsal conjunctiva, or scars on the other parts of the conjunctiva

C2 Moderate: more severe scarring but without shortening or distortion of the upper tarsus.

C3 Severe: scarring with distortion of the upper tarsus.

C6 Not applicable

- 1. Conjunctival scarring if previous surgery

SCO No scarring on the conjunctiva

SC1 Surgical line only.

SC2 Surgical line and occasional scattered scars

SC3 Surgical scar with widespread trachomatous scarring but no distortion

SC4 Surgical scar with distortion immediately around the incision line.

SC5 Surgical scar with additional distortion secondary to widespread trachomatous scarring.

SC6 Not applicable

- 1. **Shallowing/loss of the lower fornix: No/yes**
  2. **Photograph tarsal plate**
  3. **Any other examination notes**

**Study** Record Form BASELINE Trial 1 and 2

| Demographic | |
| --- | --- |
| - 1. Study reference number |  |
| - 1. Recruitment Location |  |
| - 1. Recruitment Date |  |
| - 1. First Name |  |
| - 1. Father’s Name |  |
| - 1. Grandfather’s Name |  |
| - 1. Household Head’s Name |  |
| - 1. Husband’s Name (if female + married) |  |
| - 1. Gott |  |
| - 1. Kabele |  |
| - 1. Woredah |  |
| - 1. Telephone number (if possible) |  |
| - 1. Who’s tel is this |  |
| - 1. Other demographic notes |  |

|  | **Answer Options** | **Answer** |
| --- | --- | --- |
| - 1. Sex | 1 = Male  2 = Female |  |
| - 1. Age | Years |  |
| - 1. Date of Birth (Ethiopian) | Date/Month/Year (approx if unknown) |  |
| - 1. Ethnic Group | *Coding to be determined in Ethiopia* |  |
| - 1. Literacy | 0 = Illiterate  1 = Able to read Amharic only  2 = Able to read English only  3 = Able to read other language only (specify)  4 = Able to read English and Amharic/other  5 = Refused to answer |  |

| **General Examination** | | |
| --- | --- | --- |
| - 1. Height |  | |
| - 1. Weight |  | |
| - 1. Trial eye |  | |
| - 1. Eye being treated | 1=Right  2=Left  3=Bilateral |  |

| Ophthalmic Examination (Trial patients only) | | | | | | |
| --- | --- | --- | --- | --- | --- | --- |
|  | **Answer Options** | **Right** | | **Left** | | |
| - 1. Logmar Visual Acuity (4m if possible) | Best Corrected  Pinhole | Line/ Letters | Dist | | Line/ Letters | Dist |
|  |  | / |  | | / |  |
|  |  | / |  | | / |  |
| - 1. No. of medial globe lashes |  |  | | |  | |
| - 1. No. of lateral globe lashes |  |  | | |  | |
| - 1. No. of corneal lashes |  |  | | |  | |
| - 1. Trichiasis grade | T 0,1,2,3 |  | | |  | |
| - 1. Number of metaplastic lashes |  |  | | |  | |
| - 1. Number of misdirected lashes |  |  | | |  | |
| - 1. Lower lid trichiasis | 0 = No  1 = Yes |  | | |  | |
| - 1. Entropion grade | E 0,1,2,3,4 |  | | |  | |
| - 1. Epilation | 0 = No  1 = Yes |  | | |  | |
| - 1. Epilation; how much | 0 = none  1 = <1/3rd  2 = 1/3^rd^-2/3^rd^  3 = >2/3rd |  | | |  | |
| - 1. Epilation; how successful | 0 = no epilation  1 = successful  2 = <6 lashes  3 = 6+ lashes |  | | |  | |
| - 1. Photo TT | 0 = No  1 = Yes |  | | |  | |
| - 1. Plica semilunaris | 0 = Normal  1 = Diseased but present  2 = Effaced |  | | |  | |
| - 1. Symblepharon (upper or lower) | 0 = No  1 = Yes |  | | |  | |
| - 1. Ocular discharge | 0 = No  1 = Serous (watery)  2 = Purulent  3 = Other (describe) |  | | |  | |
| - 1. Corneal scar grade | CO 1,2a,2b,2c,2d,3,4 |  | | |  | |
| - 1. Photo cornea | 0 = No  1 = Yes |  | | |  | |
| **Perform bacteriology swab – see below for coding** | | | | | | |
| - 1. Lagophthalmos | 0 = No  1 = Yes |  | | |  | |
| - 1. Conjunctivilisation of margin grade | CM 0,1,2,3 |  | | |  | |
| - 1. Follicle grade | F 0,1,2,3 |  | | |  | |
| - 1. Papillary grade | P 0,1,2,3,4,5,6 |  | | |  | |
| - 1. Conj scar grade | C 0,1,2,3,6(n/a) |  | | |  | |
| - 1. Conj scar grade if prev surgery | SC 0,1,2,3,4,5,6(n/a) |  | | |  | |
| - 1. Shallowing/loss of lower fornix | 0 = No  1 = Yes |  | | |  | |
| - 1. Tarsal plate photo | 0 = No  1 = Yes |  | | |  | |
| - 1. Other examination Notes | ***Right*** | ***Left*** | | | | |
| - 1. Swab conj bacteria | 0 = No  1 = Yes |  | | |  | |
| - 1. Bacteriology Swab No. |  |  | | |  | |
| - 1. Swab RNA | 0 = No  1 = Yes |  | | |  | |
| - 1. RNA Swab Number |  |  | | |  | |
| - 1. Swab DNA | 0 = No  1 = Yes |  | | |  | |
| - 1. DNA Swab Number |  |  | | |  | |
| - 1. Shirmer’s 1 (pre anaesthetic) | mm in 5 minutes |  | | |  | |
| - 1. Shirmer’s 2 (post anaesthetic) | Mm in 5 minutes |  | | |  | |

| Ophthalmic Questionnaire (Trial patients only) | | | | | |
| --- | --- | --- | --- | --- | --- |
|  | | **Answer Options** | **Right** | | **Left** |
| - 1. Have you ever had eye surgery | | 1 = No  2 = Trachoma surgery  3 = Non trachoma surgery (specify what surgery) |  | |  |
| - 1. If yes, what surgery | | Free text |  | |  |
| - 1. Do you have any eye pain? | | 0 = No  1 = Yes |  | |  |
| - 1. If you do have eye pain, how often do you experience this? | | 0 = Never  1 = Once a week  2 = Once a day  3 = Several times a day  4 = Constantly |  | |  |
| - 1. Do you eyes water | | 0 = Never  1 = Occasionally  2 = Often |  | |  |
| - 1. During a typical day in the past week, how often did your eyes feel dry (so that you wanted to put water or other lubricant in them) | | 0 = Never 1 = Infrequently 2 = Frequently 3 = Constantly 4 = Not sure |  | |  |
| - 1. Do you epilate? | | 0 = No  1 = Yes |  | |  |
| - 1. How often do you epilate? | | 0 = Never  1 = More than once a week  2 = once/week to once/month  3 = Less than once a month |  | |  |
| - 1. When did you last epilate? | | 0 = Never  1 = Within last week  2 = 1 week to 1 month ago  3 = Greater than 1 month ago |  | |  |
| - 1. Who does the epilating? | | 0 = never  1 = You  2 = A friend or relative  3 = Health care worker |  | |  |
| - 1. What do you epilate with | | 0 = Don’t epilate  1 = Locally made forceps  2 = Machine made forceps  3 = Burning  4 = Cutting |  | |  |
| - 1. Has a health-worker previously told you that need eyelid surgery | | 0 = No  1 = Yes |  | |  |
| - 1. When was the last time you were offered surgery? | | 0 = Never  1 = Within last year  2 = >1 year ago |  | |  |
| - 1. If surgery has been recommended or offered to you in the past, but you have not received or accepted the surgery, what are your main reasons for this? *(list as many as appropriate)* | 0 = Never offered surgery (i.e. n/a) | | | *Yes No* | |
|  | 1 = No symptoms | | | *Yes No* | |
|  | 2 = Symptoms present, but didn’t know treatment needed | | | *Yes No* | |
|  | 3 = Cost | | | *Yes No* | |
|  | 4 = No one to accompany | | | *Yes No* | |
|  | 5 = Lack of time | | | *Yes No* | |
|  | 6 = Fear of the operation | | | *Yes No* | |
|  | 7 = Resistance of family | | | *Yes No* | |
|  | 8 = Transport difficulties | | | *Yes No* | |
|  | 9 = Don’t know where to get it | | | *Yes No* | |
|  | 10 = Other (specify reason) | | | *Yes No* | |
| Of the above reasons which is:   - 1. the most important*:* | |  | | | |
| - 1. the 2^nd^ most important | |  | | | |

**I certify that all the demographic questions are completed_______________ __ Date_________**

**I certify that all the questionnaire questions are completed______________ Date_________**

**I certify that all the examination findings are recorded__________________ Date_________**

**I certify that all the answers are accurately entered on the database_______ Date_________**

**I certify that all the answers are accurately entered on the database_______ Date_________**

**Appendix C – Randomisation procedure**

**Randomisation trial 2**

**A. Preparing the master randomisation code**

- Each surgeon operating on patients in this trial will have their own separate randomisation sequence.
- Blocked randomisation codes to be generated by Helen Weiss using STATA
- The size of the blocks will vary at random.

1. Surgeon 1’s randomisation envelope sequence will be marked as follows E1/0001-E1/1000
2. Surgeon 2’s randomisation envelope sequence will be marked as follows E2/0001-E2/1000
3. Surgeon 3’s randomisation envelope sequence will be marked as follows E3/0001-E3/1000
4. Surgeon 4’s randomisation envelope sequence will be marked as follows E4/0001-E4/1000
5. Surgeon 5’s randomisation envelope sequence will be marked as follows E5/0001-E5/1000

Helen Weiss to keep one copy of the master code sheet. 2 further copies to be sent to people not involved in the trial for safe keeping.

A 4^th^ copy to be sent to a person in Ethiopia independent of the trial for the preparation of the individual envelopes.

**B. Preparation of envelopes from code sheet – to be carried out by a person independent of the trial.**

1. Prepare five boxes that can each hold 1000 envelopes. Mark the boxes Surgeon 1, 2, 3, 4, 5.
2. Use a different size or colour of envelope for each sequence to prevent mixing up.
3. Following the master code sheet do the following:
   1. Print the unique envelope number on the outside, using a different print colour for each surgeon to reduce risk of mis-allocation.
   2. Print the randomisation number and the allocation (surgery or epilation) for this number on a piece of paper and place in the envelope, with the same randomisation number on the outside.
   3. Do NOT seal the envelope.
4. In batches of 10 double check that each envelope contains the correct card. Then seal.

**C. In the field – to be performed by dedicated trained randomisation field worker**

1. Randomisation field worker will be working in location not visible to other project team members.
2. After consent and examination, the patient is sat in a waiting area.
3. When a surgeon is available for operating, the surgeon finds the randomisation field worker and together they take an envelope from that surgeon’s pile. The will open the envelope and record the instruction on masterlist E2 (see below). This will be recorded on two copies of masterlist two, with one copy being kept by the randomisation field worker and the second copy being given to a person independent of the trial. A new masterlist E2 page will be started on each trial day.
4. If the instruction in the envelope is for surgery the randomisation field worker and the surgeon take the patient through to the theatre.
5. Field worker will take 3 (if unilateral surgery required) or 6 (if bilateral) silk sutures from the suture store and give to the surgeon. They will ask the surgeon to double check that this is the correct (silk) suture
6. The surgeon will record the details of the operation in masterlist E3 (see below) to record the suture type, complications and whether the patient needs suture removal to be performed in 7-10 days. This will be recorded on two copies of masterlist E3 (2 logbooks).
7. The randomisation field worker will photocopy masterlist E3 at the end of each day and highlight all patients requiring suture removal. This highlighted list will be given to the suture removal nurse.

Master list E2 – to be recorded in book 3 ‘trial 2 treatment choice book’

| Date | Study Number | Name | Patient unique identifier | Randomisation Envelope | Treatment given | Field worker signature | Surgeon signature |
| --- | --- | --- | --- | --- | --- | --- | --- |
|  | 1 | XX |  | E1/0001 | E |  |  |
|  | 2 | Xx |  | E2/0001 | S |  |  |
|  | 3 | Xx |  | E3/0001 | E |  |  |
|  | 4 | Xx |  | E1/0002 | E |  |  |
|  | 5 | Xx |  | E3/0002 | S |  |  |
|  | 6 | Xx |  | E2/0002 | S |  |  |

Masterlist E3 – to be recorded in trial 2 ‘surgeons log-book’

| Unique identifier | Name | Date of surgery | Suture type | Complications | Surgeon number | Surgeon signature | Date for suture removal | Sutures successfully removed  (yes or no) | Signature of suture remover |
| --- | --- | --- | --- | --- | --- | --- | --- | --- | --- |
|  |  |  |  |  |  |  |  |  |  |
|  |  |  |  |  |  |  |  |  |  |
|  |  |  |  |  |  |  |  |  |  |

**Breaking the code**

Only to be done for an individual patient by Tanzi Edwards, on the instructions of the trial steering committee.

**Appendix D – The Trabut Surgical Technique**

Summary of method (as per Final Assessment of Trichiasis Surgeons, WHO 2005)

1. Refer to the WHO yellow manual for aseptic technique and administration of local anaesthetic.

2. Application of traction suture. The lid should not be everted at the start. Approximately 3 mm from the upper lid margin, insert the needle with suture through the skin and orbicularis, starting either laterally or medially. Take two large bites, about 5 mm in length, with a similar space in between. There should be a loop in the middle.

3. Everting the lid and keeping it in position. Sling the suture loop into a Trabut entropion plate or Wilde’s entropion forceps, pulling the two suture ends and at the same time everting the lid. Use the forceps as a fulcrum as the lid is everted. The lid is kept in the everted position by clamping the suture to the drape using the haemostat forceps.

4. Incision of tarso-conjunctiva. Using the blade, scratch an incision along the Arlt’s line (2–3 mm from the margin) and deepen the cut until the orbicularis muscle is reached (through the tarsal plate). Extend the incision over the whole length of the tarsal plate, ending just before the lachrymal punctum medially and laterally at the canthus. Place a tissue forceps on the proximal tarsal conjunctiva to hold the incised tarso-conjunctiva, and complete the incision with scissors.

5. Blunt dissection of the tarso-conjunctiva. Pick up the tarso-conjunctiva with tissue forceps. Insert closed scissors and spread them, dissecting the tarso-conjunctiva from the overlying orbicularis muscle. The dissection should extend for approximately 8 mm.

6. Suturing to achieve eversion of the distal fragment of the lid margin. Use mattress sutures, taking 1-mm bites of tarsal conjunctiva and half the thickness of the tarsal plate, passing the needle under the distal tarsal conjunctiva and emerging through the skin about 3 mm above the lid margin. A minimum of three or four evenly spaced mattress sutures are to be applied. To finish, all the sutures are pulled up together so as to bury the proximal fragment edge of the tarso-conjunctiva under the distal fragment. Starting in the middle, tie the sutures snugly with three single knots, and cut 3 mm above the knot.

7. Remove the traction suture and the Wilde’s entropion forceps or Trabut plate. Apply topical antibiotic and dressing as described for BLTR.

**Appendix E – The sterilisation of instruments (as per The WHO Manual for Trichiasis Surgery for Trachoma)**

Sterilization is defined as the destruction of all viruses, bacteria and spores.

1. Following surgery wash all instrument in water containing detergent. Scrub them thoroughly paying particular attention to moving part, to ensure all blood is removed.
2. Soak these washed instruments in a second bowl containing water with bleach
3. Rinse all instruments in fresh water.
4. Air dry
5. Oil instruments where appropriate
6. Prepare drums for autoclave,
7. Sterilise the drums in pressure cooker autoclave for more than 15 minutes at 121 °C, 101 KPa, accoding to the autoclave manufacturer’s instructions.

**Appendix F – Diagrammatic Representation of Corneal Scar Grading**

**Appendix G:**

**Data Safety Monitoring Committee – terms of reference:**

A Data Safety Monitoring Committee (DSMC) will be responsible for monitoring the safety of this trial.

***Specific terms of reference****:*

1. Protect the safety of the study participants.
2. Review research protocol and trial documentation.
3. To determine if an interim analysis of trial data should be undertaken.
4. To determine what should be reported to the DSMC as a serious adverse event (SAE).
5. To consider the data from interim analyses (if deemed necessary), unblinded if considered appropriate, plus any additional safety issues for the trial and relevant information from other sources.
6. In the light of 5., to report to make recommendations to the Trial Steering Committee regarding modifications or continuation of the trial.

***Membership****:*

There will be 3 members, all independent of the running of the trial with relevant clinical and epidemiological experience. They will be appointed by the PI.

***Meeting schedule****:*

The DSMC will determine their specific meeting arrangements. It is proposed that the DSMC would meet prior to the beginning of the trial, half way through (one year) and at the end (two years).

***Quorum****:*

2 members

***Data monitoring and interim analyses:***

The DSMC will determine the manner in which it will monitor the data, what it requires from the investigators in this respect and will communicate this to the PIs. Similarly it will determine the need for interim analyses and undertake these in a manner which does not compromise the integrity of the trial.

***Serious Adverse Event reporting****:*

The process for and boundaries around SAE reporting will be determined by the DSMB and the PI advised accordingly. Reporting will be to the Safety Monitor (a designated member of the DSMC) who will report to the DSMC as appropriate.

**Appendix H:**

**Glossary**

Dystichiasis

A congenital aberrant row of lashes.

Entropion

Inwards rotation of the lid margin.

Lid margin

The free margin of the eyelid. In the normal eye this is approximately 2mm thick and square in cross section, except in the medial one-sixth (medial to the punctum) where it is rounded. The outer boundary of the lid margin is the lash base line of the inner most row of eyelashes. The lid margin is covered in skin continuous with the outer lid and contains meibomian gland openings in an orderly row. The inner margin is where the skin ends, just prior to where the lid touches the cornea in primary position and conjunctiva begins.

Metaplastic lashes

Acquired aberrantly located eyelashes. These often arise from meibomian gland orifices.

Misdirected lashes

Lashes that point in an abnormal direction, but whose base is in a normal position.

Plica Semilunaris

A semi-lunar fold of conjunctiva lying on the lateral side and partly underneath of the caruncle. In the normal eye, the lateral margin is free and concave. Beneath the fold is a small space about 2mm deep when the eye is looking medially. When the eye is looking laterally, the space practically disappears.

**Studies on the Management of Trachomatous Trichiasis**

**Analytical Plan for Trial 2: Randomized controlled trial of immediate surgery vs. epilation for Minor Trichiasis**

**1. Participant flow**

*a) Eligibility, enrolment and follow-up*

The following will be shown by trial arm in a flowchart following CONSORT guidelines for non-inferiority trials (JAMA 2006: 295:1152-1160): Numbers eligible, consenting to take part, randomized, and who received the intended treatment. The numbers still in follow-up, censored, defaulting, and permanently lost-to-follow-up respectively at each visit will also be shown by arm. Reasons for declining to take part, not having surgery, or discontinuing follow-up will be summarized by arm.

*b) Censoring*

Patients in both arms will be considered as failures and immediately censored when they present with 5+ lashes in the trial eye. In addition, patients who present having had surgery (or repeat surgery) on the trial eye from other providers will be considered as failures and immediately censored.

1. **Description of baseline data**

The following characteristics of participants at baseline will be tabulated by arm:

- 1. Socio-demographic factors: age, sex, literacy, ethnic group.
  2. Behavioural factors: epilation habits, whether surgery has previously been offered, reasons for not having surgical treatment previously.
  3. Clinical status: trichiasis, entropion, inflammation, conjunctivilisation of lid margin, corneal scarring, evidence of epilation, visual acuity, symblepharon.

The distributions of these variables by treatment arm will be compared, to assess whether there is imbalance at baseline in these potential confounding factors.

1. **Primary outcome**

*a) Definition*

The primary outcome measure will be the proportion of participants with 5 or more lashes touching the eye on one or more follow-up visits. Lids with eyelash stubs which are not in contact with the eye will not be considered a treatment failure.

*b) Justification of primary outcome measure*

Trichiasis (lashes touching the eyeball) causes visual loss through corneal opacification. The purpose of the interventions being compared in this trial is to stop eyelashes touching the eyeball. It is anticipated that some of the patients in the surgery arm of the trial may commence epilation if their surgery fails. From an ophthalmic public health perspective they are ‘successes’ if they successfully prevent eyelashes touching the globe, despite the surgery itself having not been totally successful.

Previous studies have used a cut-off of 5 or more lashes for determining treatment choices (Arch Ophth 2006; 124: 309-314). Similarly, this level has been used in national blindness control programmes (The Gambia) as an indication for surgery.

*c) Sample size*

Our sample size of 1300 trial participants would provide 90% power to detect non-inferiority, assuming 10% loss to follow-up over 2 years, a 5% failure rate in the surgery arm, and non-inferiority margin of 10%. (i.e we would conclude non-inferiority if the upper 95%CI for the observed difference in failure rates was less than 10%)

The power to detect non-inferiority under different assumptions with this sample size is shown in the table below

**Table 1:** Power for detecting non-inferiority under different assumptions with sample size of 585 per arm (1300 recruited, 10% loss to follow-up)

| **Proportion failing in surgery arm (**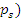 | **Proportion failing in epilation arm (**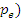 | **Difference in proportions (**µ) | **Inferiority margin (**Δ) | **Power** |
| --- | --- | --- | --- | --- |
| 2% | 6% | 4% | 8% | 93% |
| 2% | 8% | 6% | 10% | 89% |
| 3% | 6% | 3% | 8% | 98% |
| 3% | 9% | 6% | 10% | 85% |
| 4% | 8% | 4% | 8% | 82% |
| 4% | 8% | 4% | 10% | 99% |
| 4% | 9% | 5% | 10% | 94% |
| 5% | 9% | 4% | 8% | 76% |
| **5%** | **10%** | **5%** | **10%** | **90%** |
| 8% | 11% | 3% | 8% | 83% |
| 8% | 12% | 4% | 10% | 93% |

*d) Statistical analysis*

Statistical analyses will calculate the difference in proportion failing in the two arms, and 95%CI for this difference using exact methods. Non-inferiority of epilation will be shown if the upper two-sided 95%CI around the estimated difference in proportions is below 10%. Blinded analyses conducted at the end of the trial showed an overall failure rate of 8%. Table 2 shows conclusions for different assumptions of the proportion failing in the surgery and epilation arm respectively. Given an overall failure rate of 8%, the conclusion will be that epilation is non-inferior to surgery if the proportion failing in the surgery arm is greater than 4.5%. Epilation will be deemed inferior, if the failure rate in the epilation arm is 15% or higher.

**Table 2: Examples of results, under different observed results**

| **Proportion failing in surgery arm** | **Proportion failing in epilation arm** | **Difference in proportions** | **95% CI for difference** | **Conclusions** |
| --- | --- | --- | --- | --- |
| 0% | 16% | 16% | 13.1%-19.0% | Inferior |
| 1% | 15% | 14% | 11.0%-17.0% | Inferior |
| 2% | 14% | 12% | 9.0%-15.0% | Inconclusive |
| 3% | 13% | 10% | 6.9%-13.1% | Inconclusive |
| 4% | 12% | 8% | 4.9%-11.1% | Inconclusive |
| 4.5% | 11.5% | 7% | 3.9%-10.1% | Borderline non-inferior |
| 5% | 11% | 6% | 2.9%-9.1% | Non-inferior |
| 6% | 10% | 4% | 0.9%-7.1% | Non-inferior |
| 7% | 9% | 2% | -1.1%-5.1% | Non-inferior |
| 8% | 8% | 0% | -3.1%-3.1% | Non-inferior |

*e) Primary analysis*

Our primary analysis will be a modified intention to treat (ITT) analysis, which will include only those individuals who were seen at least once.

*Justification for using modified ITT:*

In a trial comparing two drug treatments a per-protocol analysis would be used to try to determine the ‘true’ effect of the drug treatment (excluding for example issues of drug compliance), by only analysing the participants who follow their treatment allocation. However, it is not appropriate in the present study, which compares a one-off treatment (surgery), in which all participants follow their treatment allocation, with a repeated treatment (epilation), in which participants may not adhere. Therefore, a per-protocol analysis would require the comparison of all participants in one arm with a biased selection from the other arm. The per-protocol analysis would require exclusion of individuals not epilating in the epilation arm. However, some of these individuals may have epilated once or twice and found the lashes not to recur, thus obviating the need to epilate. These people are treatment successes and it would be wrong to exclude them from the analysis.

A standard intention to treat (ITT) analysis includes all individuals whether or not they adhered to treatment, i.e. all individuals are included in the analysis irrespective of whether they were followed-up. The benefit of an ITT is that it eliminates potential bias of differential loss to follow-up. However, in a non-inferiority trial, failure to follow people up, biases towards non-inferiority and type I error. Therefore, in order to prevent bias towards non-inferiority, the primary analysis included only those individuals who were seen at least once, i.e. a modified intention-to-treat analysis.

*f) Secondary analyses*

1. **Time-to-first-failure:** The time-to-first-failure will be analysed with Cox regression analysis, comparing failure rates in the two trial arms.

*g) Subgroup analyses*

The effect of the intervention on failure will be stratified by presence of:

1. Entropion at baseline
2. Conjunctivilisation at baseline

For each analyses, we will test for effect-modification by strata

**Secondary outcomes**

1. *Number of lashes touching the eye at each visit.*

This will include data from all time points. We expect a large proportion of individuals with no lashes touching, and will thus use a random effects zero-inflated Poisson analysis (Lee et al, Statistical Methods in Medical Research 2006:15:47-61). This model is suitable for count data with an excess of zeros, and adjusts for within-patient correlation. The model will provide a coefficient, 95%CI and p-value for the difference in log mean number of lashes in the intervention versus control arms.

1. *Lashes touching the cornea:*

The number of lashes touching just the cornea will be compared between the two arms of the trial. Lashes that only touch the peripheral eyeball (i.e. the sclera rather than the cornea), are probably not of great visual or pathological significance. This will be analysed with similar methods to that for the total number of lashes touching the eye at each visit.

1. *Entropion grade*

The effect of the intervention on the degree of entropion at 24 months will be analysed by ordered logistic regression.

1. *Conjunctivilisation of the lid margin grade*

The effect of the intervention on the degree of conjunctivilisation at 24 months will be analysed by ordered logistic regression.

1. *Corneal opacification*
   1. Descriptive measures of the baseline severity of CO
   2. Description of the change in CO between baseline and one and two year follow up time-points.
   3. Ordinal logistic regression analysis for change in CO category at one year and two years respectively, to identify factors associated with it, such as inflammation.
   4. The effect of the intervention on the degree of change in CO between baseline and 24 months will be analysed by ordered logistic regression.
2. *Visual acuity*

The effect of the intervention on the visual acuity (logMAR score) at 12 and 24 months will be analysed by linear regression.

1. *Surgical complications and recurrence rate (in surgical arm)*

Surgical complications such as granuloma, incomplete lid closure, inflammation from suture material and infection can occur. The clinical status of the eyelid at the suture removal (7-10 days) will be described. The risk of complications in the surgical arm of the trial will be calculated at one year and two years respectively. The risk of recurrence (defined as at least one lash touching the eyeball or clinical evidence of epilation or redo surgery) will also be estimated at each visit.

1. *Patient satisfaction*

Patients are asked the following questions on the follow up questionnaires that indicate their satisfaction with the procedure:

- 1. *Do you think your vision is worse, the same or better than before your surgery or epilation?*
  2. *Do you have eye pain?*
  3. *If you have eye pain is it worse, the same or better than before your surgery or epilation?*
  4. *Do your eyes water?*
  5. *If you had surgery, did you find it painful?*
  6. *If you have been epilating do you find it painful?*
  7. *If you had surgery and the eyelashes have returned, do you want to have further surgery?*
  8. *If you have been epilating would you prefer to have had surgery?*

1. The answers to each of these questions will be compared between the two arms of the trial at 12 months. Additionally the answers to questions (b) and (d) will be adjusted for baseline (pre-operative) response.

Footnotes

1. *Why is corneal opacification not the primary outcome measure?*

Although corneal opacification is the outcome that is responsible for blindness (rather than trichiasis which is a risk factor for this) it can not be used as a primary measure for the following reasons:

1. As few as 10% of patients will have corneal opacity at the outset of the study.
2. The rate of change of corneal opacity can be slow, and the clinical examination is not sensitive enough to detect very subtle changes
3. Corneal opacity can be caused by other insults such as infection and trauma.
4. *Why is visual acuity not the primary outcome measure*

As with corneal opacity, loss of visual acuity is the end stage process from the disease. However it can not be used as a primary outcome measure because it is affected by many other common conditions such as cataract, as well as simply by aging.

**Summary of literature on non-inferiority for a binary outcome**

Rousson and Seifert (Biom J 2008 50(2)190-204

Let p_I_=proportion with outcome in intervention arm

p_C_=proportion with outcome in control arm

We can consider the difference of proportions D=p_I_-p_c_  or the odds ratio

The non-inferiority margin for D (Δ_D_) should depend on the value of p_I_.

The FDA (1997) recommend Δ_D_=0.1 if p_I_ > 0.1

(ref: http://www.fda.gov/downloads/Drugs/GuidanceComplianceRegulatoryInformation/Guidances/UCM202140.pdf

If one uses odds ratios, the choice of Δ is independent of p_I_ Several authors suggest an Δ_OR_ = 0.5

**Sample size**

The formula for sample size per arm when the expected proportions with the outcome differ by arm is:


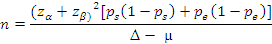


where


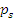
=proportion with outcome in control (surgery) arm


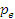
=proportion with outcome in intervention (epilation) arm

Δ = inferiority margin (value such that if the alternative hypothesis is true, the upper 95% CI for the difference in proportion failing will not exceed Δ with probability 1-
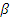


µ = expected difference in the treatment means (
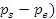


β = power

α = 0.025 (
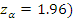

Supplement: Text S1 — Trial protocol. (DOC) [file pmed.1001136.s002.doc]
